# Crizotinib attenuates cancer metastasis by inhibiting TGFβ signaling in non-small cell lung cancer cells

**DOI:** 10.1038/s12276-022-00835-8

**Published:** 2022-08-23

**Authors:** Soonbum Park, Eun A Cho, Jung Nyeo Chun, Da Young Lee, Sanghoon Lee, Mi Yeon Kim, Sang Mun Bae, Su In Jo, So Hee Lee, Hyun Ho Park, Tae Min Kim, Insuk So, Sang-Yeob Kim, Ju-Hong Jeon

**Affiliations:** 1 Department of Physiology and Biomedical Sciences, Seoul National University College of Medicine, Seoul, Korea; 2 ASAN Institute for Life Sciences, ASAN Medical Center, Seoul, Korea; 3 Department of Medical Science, Asan Medical Center, University of Ulsan College of Medicine, Seoul, South Korea; 4 Institute of Human-Environment Interface Biology, Seoul National University, Seoul, Korea; 5 Department of Biochemistry, University of Utah School of Medicine, Salt Lake City, UT USA; 6 College of Pharmacy, Chung-Ang University, Seoul, Korea; 7 Cancer Research Institute, Seoul National University College of Medicine, Seoul, Korea; 8 Department of Internal Medicine, Seoul National University Hospital, Seoul, Korea

**Keywords:** Non-small-cell lung cancer, Targeted therapies

## Abstract

Crizotinib is a clinically approved tyrosine kinase inhibitor for the treatment of patients with locally advanced or metastatic non-small cell lung cancer (NSCLC) harboring EML4-ALK fusion. Crizotinib was originally developed as an inhibitor of MET (HGF receptor), which is involved in the metastatic cascade. However, little is known about whether crizotinib inhibits tumor metastasis in NSCLC cells. In this study, we found that crizotinib suppressed TGFβ signaling by blocking Smad phosphorylation in an ALK/MET/RON/ROS1-independent manner in NSCLC cells. Molecular docking and in vitro enzyme activity assays showed that crizotinib directly inhibited the kinase activity of TGFβ receptor I through a competitive inhibition mode. Cell tracking, scratch wound, and transwell migration assays showed that crizotinib simultaneously inhibited TGFβ- and HGF-mediated NSCLC cell migration and invasion. In addition, in vivo bioluminescence imaging analysis showed that crizotinib suppressed the metastatic capacity of NSCLC cells. Our results demonstrate that crizotinib attenuates cancer metastasis by inhibiting TGFβ signaling in NSCLC cells. Therefore, our findings will help to advance our understanding of the anticancer action of crizotinib and provide insight into future clinical investigations.

## Lung cancer: existing drug blocks cancer spread

Investigating the activity of an existing anticancer drug shows that it can limit metastasis (cancer spread) in non-small-cell lung cancer (NSCLC). Aberrant signaling from the transforming growth factor β (TGFβ) protein is known to trigger metastasis in NSCLC. The drug crizotinib is clinically approved for NSCLC, but whether it can affect metastasis is unclear. Ju-Hong Jeon (Seoul National University College of Medicine), Sang-Yeob Kim (ASAN Medical Center, Seoul) and co-workers used cell cultures and a mouse model to examine if crizotinib can inhibit TGFβ. Crizotinib suppressed TGFβ signaling by blocking the phosphorylation of a critical protein and inhibiting the enzymatic activity of a TGFβ receptor. These actions reduced the cell migration and invasion usually mediated by TGFβ. The results also indicate that cancer signaling pathways can act independently, meaning that a multitarget approach may improve treatment.

## Introduction

Tumor metastasis is the end result of a series of cell biological events that enable cancer cells to disseminate from primary tumors and survive in the new tumor microenvironment in distant tissues^1,2^. Since most metastatic cancers are resistant to conventional therapeutic agents, tumor metastasis is the major cause of mortality in patients with cancer, including non-small cell lung cancer (NSCLC), which therefore remains a major clinical challenge^3,4^. Therefore, targeting metastasis as a strategy for the prevention or inhibition of initial or recurrent metastasis and for the treatment of established metastatic tumors holds clinical promise for patients with or at risk of metastatic cancer^5^.

During the invasion-metastasis cascade, tumor cells undergo distinct changes in signaling pathways, such as transforming growth factor β (TGFβ) and c-mesenchymal-epithelial transition factor (MET), which lead to epithelial plasticity, cell migration and invasion, and metastatic colonization^6,7^. In particular, aberrant TGFβ signaling promotes tumor metastasis by mediating the multiple steps of metastasis, such as epithelial-mesenchymal transition (EMT), cell invasion, the interaction of circulating tumor cells with platelets, and transendothelial migration, and is associated with poor therapeutic outcome and prognosis in patients with NSCLC^8–12^. Therefore, TGFβ signaling has attracted much attention as a therapeutic approach to cancer metastasis.

Crizotinib was originally developed as a MET tyrosine kinase inhibitor and was later found to inhibit anaplastic lymphoma kinase (ALK), v-ros UR2 sarcoma virus oncogene homolog 1 (ROS1), and recepteur d’origine nantais (RON) kinases^13–16^. Crizotinib demonstrated potent antitumor activity in patients with NSCLC harboring echinoderm microtubule-associated protein-like 4 (EML4)-ALK fusion or ROS1 rearrangement, which led to FDA approval for its clinical use^13–15,17^. However, emerging evidence suggests that the anticancer effect of crizotinib is largely attributable to uncharacterized off-target mechanisms^18^.

MET, the hepatocyte growth factor (HGF) receptor, is involved in the development of metastasis in various types of human cancer^19,20^, including NSCLC^21–23^. Given that crizotinib inhibits MET, these findings suggested that crizotinib can suppress tumor metastasis. In addition, recent evidence has shown that crizotinib may be beneficial for patients with metastatic EML4-ALK-positive NSCLC^24,25^. However, little is known about whether crizotinib attenuates tumor metastasis in NSCLC cells. Furthermore, the effect of crizotinib on TGFβ signaling has not been investigated in NSCLC cells.

Based on previous findings and our data-driven inference, in this study, we hypothesized that crizotinib exerts antimetastatic activity by inhibiting TGFβ signaling. We provided evidence that crizotinib suppresses tumor metastasis by directly inhibiting TGFβ receptor I (TβRI) kinase activity in NSCLC cells. Our results will enhance our knowledge about the molecular mechanisms underlying the anticancer actions of crizotinib, providing insight into the clinical usefulness of crizotinib in patients with advanced NSCLC.

## Materials and methods

### Cell culture and reagents

NCI-H3122 cells were kindly provided by Professor Pasi A. Janne (Dana Faber Cancer Institute, Boston, MA, USA). A549, Calu-1, Calu-3, H1975, H2228, PC-9, and SNU2535 cells were supplied by the American Type Culture Collection (ATCC, Manassas, VA, USA) or the Korean Cell Line Bank (KCLB, Seoul, Korea). All cell lines were confirmed to be mycoplasma free. All cell culture reagents were obtained from Gibco (Grand Island, NY, USA) or HyClone (Logan, UT, USA). Before treatment with TGFβ1 (R&D Systems, Minneapolis, MN, USA), cells were maintained in RPMI 1640 medium containing 0.2% fetal bovine serum (FBS) for 2 h. Crizotinib was purchased from Tocris Bioscience (Ellisville, MO, USA). Alectinib, ceritinib, PHA-665752, and savolitinib were obtained from Selleckchem (Houston, TX, USA). SB431542 was purchased from EMD Millipore (Darmstadt, Germany). All other reagents not specified were supplied by Sigma-Aldrich (St. Louis, MO, USA).

### DNA microarray experiments

DNA microarray experiments were performed using total RNA from A549 cells following treatment with 10 μM crizotinib for 24 h in the presence or absence of 1 ng/ml TGFβ. The microarray data are available through the Gene Expression Omnibus (GEO) database under the accession number GSE189047.

### Bioinformatic analysis

We used GSE89127 (public RNA-seq datasets of NCI-H3122 cells treated with crizotinib), GSE31210 (public microarray datasets of patients with NSCLC), and GSE189047. For GSE89127 data analysis, sequence read alignment was conducted using Rsubread (version 1.28.1), and normalization was performed using edgeR (version 3.22.3) and Limma R (version 3.36.2) packages to determine gene expression levels in log2 transcripts per million (TPM). Sequence reads were aligned to the human reference genome GRCh38, and the aligned sequences were mapped to NCBI gene IDs by using NCBI gene annotation data. For GSE31210 and GSE189047 data analysis, SCAN normalization was used to normalize gene expression levels, and microarray probe sets were mapped to 17,052 NCBI Entrez gene IDs using a custom mapping file (version 22.0.0) from the BrainArray resource. Then, we performed hierarchical clustering with Euclidean distance and principal component analysis (PCA) to verify that both datasets had distinctive gene expression profiles. We used the Linear Models for Microarray Data (Limma) Bioconductor R package to identify differentially expressed genes (DEGs). The *t* values in the Limma results were used to supply the ranked list of genes into GSEAPreranked to examine gene signature enrichment in the hallmark or canonical pathways of the Molecular Signature Database (v7.2). The significant gene signatures were determined by the threshold false discovery rate (FDR) *q* value of 0.1.

### Luciferase assay

Cells were transfected with pGL2-3TP-luciferase and pCMV-β-galactosidase gene constructs using FuGENE 6 reagent (Promega, Indianapolis, IN, USA) as previously described in ref. ^26^. After 24 h of transfection, the cells were incubated with TGFβ, crizotinib, or both for 24 h and then assayed for reporter gene activity using a commercial kit (Promega, Madison, WI, USA). The luciferase activity was normalized to β-galactosidase activity as previously described^26^.

### Western blot analysis

The crude extracts were prepared by incubation with RIPA buffer containing protease and phosphatase inhibitor cocktails (EMD Millipore, La Jolla, CA, USA). The samples were resolved using 6 or 10% SDS-PAGE and probed with the indicated antibodies. Antibodies against AKT, phospho-AKT, ALK, pALK, E-cadherin, MET, pMET, Smad3, pSmad3, S6K, p S6K, and TβRI were purchased from Cell Signaling Technology (Danvers, MA, USA). Antibodies against Myc, RON, vimentin, and fibronectin were obtained from Santa Cruz Biotechnology (Santa Cruz, CA, USA). An anti-β-tubulin antibody was supplied by Sigma-Aldrich. The signals were determined using an Amersham ECL western blotting detection reagent (GE Healthcare, NJ, USA). The data were representative of at least three independent experiments. Full-scan images of western blots are shown in Supplementary Fig. 15.

### Transfection with small interfering RNAs (siRNAs)

The siRNAs against ALK (siALK)-1 (5′-CCUGUAUACCGGAUAAUGAUU-3′), siALK-2 (5′-CCGCUUUGCCGAUAGAAUAUU-3′), siMET-1 (5′-AGAAUGUCAUUCUACAUGAGCUU-3′), siMET-2 (5′-CAUAUUCACAUUCAUCUCGGAUU-3′), siTβRI-1 (5′-CCAUCGAGUGCCAAAUGAAUU-3′), siTβRI-2 (5′-GCAUCUCACUCAUGUUGAUGGUCUAUU-3′), siRON-2 (5′-GGGCGACAGAAAUGAGAGUUU-3′), and siGFP (5′-GCAAGCUGACCCUGAAGUUCAUUU-3′) as negative control were obtained from Genolution (Seoul, Korea). siRON-1 (5′-GGGCGACAGAAAUGAGAGUUU-3′) was purchased from Santa Cruz Biotechnology. Cells were transfected with each siRNA (50 nM) using Lipofectamine RNAiMAX reagent (Invitrogen, Carlsbad, CA, USA) for 48 h and then treated with or without TGFβ for the indicated times prior to western blot analysis or luciferase assay.

### In vitro TβRI kinase activity assay

The TGFβRI Kinase Enzyme System (Promega, Madison, WI, USA) was used in this study. TβRI kinase activity was measured using an in vitro ADP-Glo kinase assay according to the manufacturer’s protocols. TβRI (20 ng/μL), TβRI substrate peptide (200 ng/μL), ATP (25–800 μM), and crizotinib (the indicated concentrations) in kinase assay buffer (Promega) were incubated for 1 h at room temperature. Luminescence was measured using a plate-reading luminometer (Tecan, Männedorf, Switzerland).

### Cell tracking assay

Cells were plated in 35 mm collagen-coated glass-bottom dishes and grown to 60% confluence. Cells were starved with RPMI 1640 containing 0.2% FBS and 1% antibiotics for 2 h and then treated with the indicated reagents, inhibitors, or both. Migrated cells were observed at intervals of 2 min for 12 h under a BioStation time-lapse imaging system (Nikon, Tokyo, Japan), and fifteen different fields were counted. Data analyses were performed with the manual tracking plugin in ImageJ software, and plots were obtained from the chemotaxis and migration tool program.

### Scratch-wound assay

A scratch-wound assay was used to assess cell migration, as previously described in ref. ^27^. Briefly, cells (1.5 × 10^5^ cells/well) were grown in six-well plates until they were fully confluent. A scratch was made on the cell monolayer using a sterile 10-μL pipette tip, and then cells were treated with the indicated reagents, inhibitors, or both in RPMI 1640 medium containing 0.2% FBS for 24 h. Migrated cells in scratched space were counted using a phase-contrast microscope (Nikon Eclipse TS100, Nikon Instruments, Inc., Melville, NY, USA). The wound closure area was calculated using ImageJ software.

### Transwell migration assay

Transwell filters (8-μm pore size, 24-well) were coated with 0.1 mg/mL collagen for 1 h. Cells (1.5 × 10^5^ cells/well) were placed in the upper transwell in a serum-free medium (100 μL). Then, TGFβ (5 ng/mL), HGF (50 ng/mL), or both of them, as chemoattractants in serum-free medium (800 μL), were placed in the lower chamber. After incubation for 24 h, migrated cells in the lower chamber were fixed with 4% formaldehyde in phosphate-buffered saline, stained with 0.2% crystal violet, and counted under a phase-contrast microscope.

### In vivo bioluminescence imaging

Calu-1-Luc cells were prepared by stable transfection with IVISbrite Red F-luc-GFP Lentiviral Particles (PerkinElmer, Waltham, MA, USA). BALB/c-nude mice (male, 8 weeks old, Charles River, Yokohama, Japan) were injected intravenously with 1 × 10^6^ Calu-1-Luc cells via the tail-vein as previously described in ref. ^28^. Vehicle (water, 200 µL) or crizotinib (with 10 or 25 mg/kg, 200 μL) was administered by oral gavage daily for 2 weeks. For in vivo bioluminescence imaging (BLI), mice were injected intraperitoneally with D-Luciferin (150 mg/kg, 200 μL, Gold Biotechnology, St. Louis, MO) and imaged 10 min later using the IVIS spectrum system (PerkinElmer, Hopkinton, MA USA). Ex vivo BLI was performed on the organs isolated from mice after the last in vivo BLI. BLI intensity was measured using region of interest (ROI) analysis. All animal experiments were performed in accordance with protocols approved by the Institutional Animal Care and Use Committee (IACUC) of the Asan Institute for Life Sciences at the Asan Medical Center (approval no. 2020‑13‑122).

### Immunofluorescence staining analysis

Immunofluorescence staining of metastatic tumor tissues was performed using a Leica Bond Rx™ Automated Stainer (Nussloch, Germany), and the images were analyzed using a Vectra Polaris Automated Quantitative Pathology Imaging System and inForm Image Analysis software (Akoya Biosciences, CA, USA) as previously described in ref. ^29^. Tissue slides were labeled with anti-pSmad3 antibody (Cell Signaling Technology) and goat anti-rabbit IgG HRP polymer (Abcam, Cambridge, UK) and probed with Tyramide Signal Amplification (Akoya Biosciences) and DAPI (Thermo Fisher Scientific, Dreieich, Germany).

### Tumor xenograft experiments

BALB/c-nude mice (male, 5 weeks old) were subcutaneously inoculated with Calu-1 cells (1 × 10^7^ cells per mouse) in the right flank of each mouse. When the tumor reached approximately 70–90 mm^3^, the mice were randomly divided into three groups (five in each group) and orally administered crizotinib (0, 25, or 100 mg/kg) every other day for 22 days. The tumor size was assessed every 2 days. The mice were sacrificed after 22 days, and tumors were excised to measure volume and weight. These animal experiments were performed in accordance with protocols approved by the Institutional Animal Care and Use Committee of the Asan Institute for Life Sciences at the Asan Medical Center (approval no. 2021-13-165).

### Statistical analysis

A comparison of mean values among experimental groups was performed using one-way ANOVA followed by a post hoc test. *p* < 0.05 was considered statistically significant.

## Results

### Crizotinib specifically inhibits TGF**β** signaling by blocking Smad3 phosphorylation

To infer clinically significant latent relationships between drug-cell signatures and disease signatures, we investigated the gene expression signatures of the GSE89127 (EML4-ALK-positive NCI-H3122 NSCLC cells treated with crizotinib) and GSE31210 (patients with EML4-ALK-positive NSCLC) datasets (Supplementary Fig. 1a–d). From 1697 DEGs that showed anti-similarity (or inverse correlation) between these two datasets (Supplementary Fig. 1e, f), we identified EMT as a clinically significant hallmark signature; the EMT signature was enhanced in patients with NSCLC bearing EML4-ALK, whereas it was decreased by crizotinib (Table 1 and Supplementary Fig. 2). Based on the fact that TGFβ is a major mediator of EMT^30,31^, we hypothesized that crizotinib inhibits TGFβ signaling in EML4-ALK-positive NSCLC cells.

**Table 1:** List of clinically significant crizotinib-induced gene signatures in NCI-H3122 cells.

Hallmark signature	ES^a^	NES^b^	NOM *p* val^c^	FDR *q* val^d^
HALLMARK_MTORC1_SIGNALING	−0.656	−2.999	0.000	0.000
HALLMARK_GLYCOLYSIS	−0.599	−2.687	0.000	0.000
HALLMARK_MYC_TARGETS_V1	−0.525	−2.310	0.000	0.000
HALLMARK_EPITHELIAL_MESENCHYMAL_TRANSITION	−0.578	−2.228	0.000	0.002
HALLMARK_MYC_TARGETS_V2	−0.603	−2.121	0.000	0.002
HALLMARK_KRAS_SIGNALING_UP	−0.504	−2.022	0.002	0.006
HALLMARK_COAGULATION	−0.594	−2.013	0.002	0.006
HALLMARK_UNFOLDED_PROTEIN_RESPONSE	−0.499	−1.951	0.004	0.009

^a^Enrichment score.

^b^Normalized enrichment score.

^c^Nominal *p* value.

^d^False discovery rate *q* value.

**Fig. 1:**
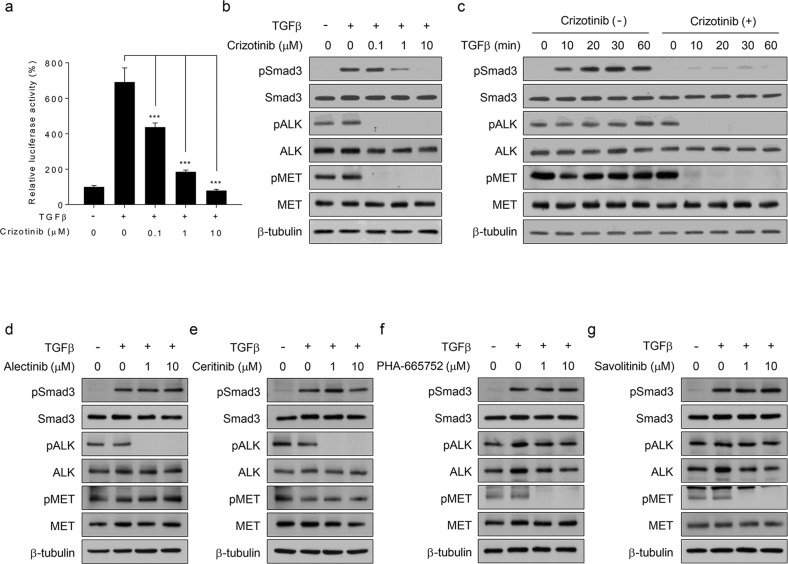
Crizotinib blocks TGFβ-induced Smad3 phosphorylation. **a** NCI-H3122 cells were transfected with a pGL2-3TP-luciferase plasmid for 24 h and further incubated with TGFβ (1 ng/mL), crizotinib (the indicated concentrations), or both for 24 h. Luciferase activity was expressed as a relative value compared to that of the untreated cells, which was set to 100%. The data were expressed as the mean ± SEM (*n* = 6). ***p < 0.005. **b**, **c** NCI-H3122 cells were treated with TGFβ (1 ng/mL), crizotinib (the indicated concentrations or 10 μM), or both for 30 min or the indicated times prior to western blot analysis. **d**–**g** NCI-H3122 cells were treated with TGFβ (1 ng/mL), ALK-specific inhibitors (alectinib and ceritinib, 1 or 10 μM), and MET-specific inhibitors (PHA-665752 and savolitinib, 1 or 10 μM), or two of them for 30 min prior to western blot analysis.

To confirm this data-driven hypothesis, we first performed reporter gene assays in EML4-ALK-positive NCI-H3122 cells. Crizotinib inhibited TGFβ-mediated luciferase activity in a dose-dependent manner (Fig. 1a and Supplementary Fig. 3). Western blot analysis showed that crizotinib abolished the phosphorylation of Smad3 in TGFβ-treated NCI-H3122 cells without affecting its total expression level (Fig. 1b). The time course analysis also showed that crizotinib blocked TGFβ-induced Smad3 phosphorylation (Fig. 1c). These results indicate that crizotinib inhibits TGFβ signaling by blocking Smad3 phosphorylation.

To determine whether the results from Fig. 1a–c are specific to crizotinib, we repeated the abovementioned experiments with other ALK-specific inhibitors (alectinib and ceritinib) or MET-specific inhibitors (PHA-665752 and savolitinib) in NCI-H3122 cells. Western blot analysis showed that none of the inhibitors used abolished TGFβ-induced Smad3 phosphorylation (Fig. 1d–g). Under these assay conditions, we confirmed that the ALK- or MET-specific inhibitors suppressed the phosphorylation of only their own targets (Fig. 1d–g), whereas crizotinib simultaneously inhibited both ALK and MET activity (Fig. 1b, c). Altogether, our results demonstrate that crizotinib specifically suppresses TGFβ signaling by blocking Smad3 phosphorylation. However, it is unclear whether ALK or MET mediates the inhibitory effect of crizotinib on TGFβ signaling.

### Crizotinib inhibits TGFβ signaling in an ALK/MET/RON/ROS1-independent manner

**Fig. 2:**
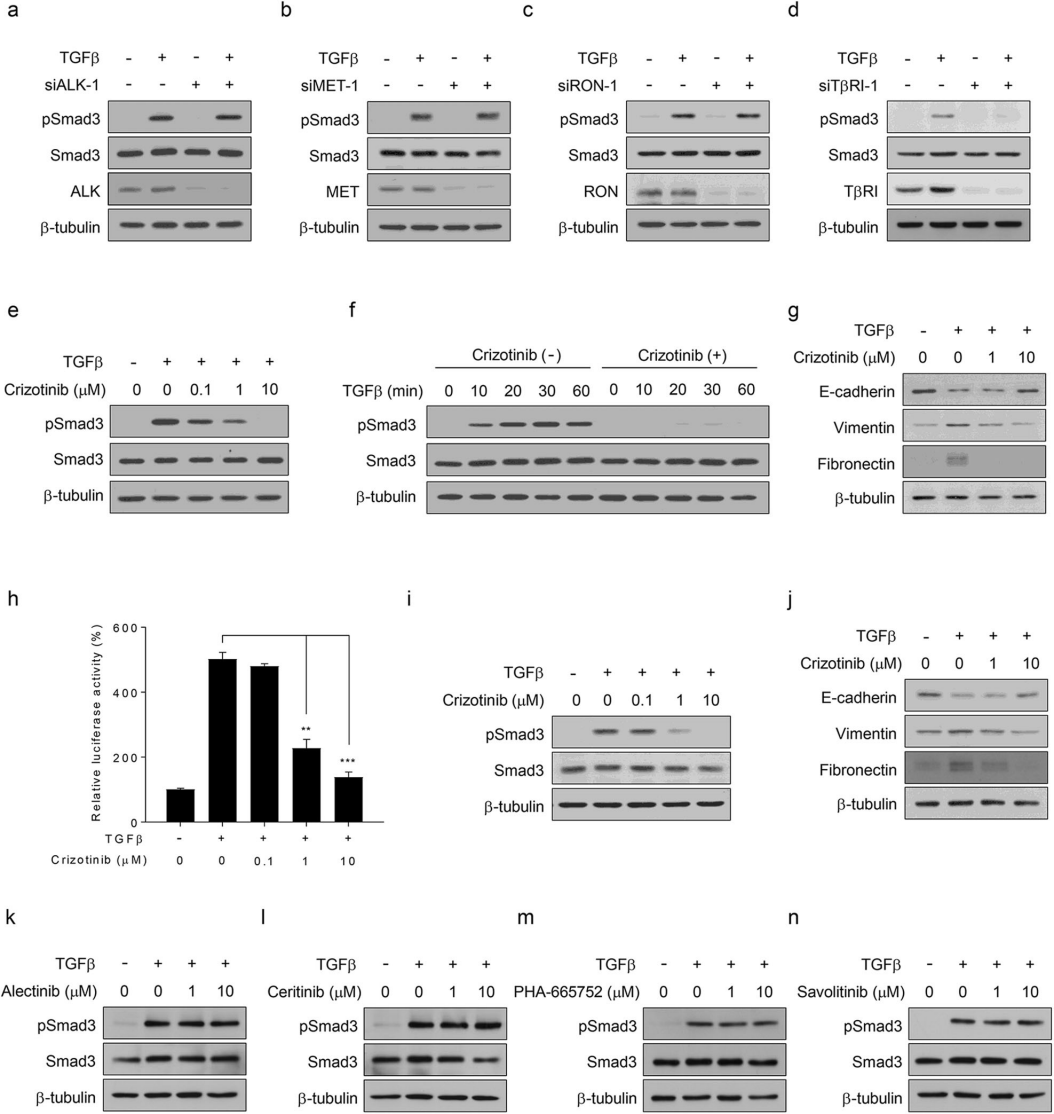
Crizotinib inhibits TGFβ signaling in an ALK/MET/RON/ROS1-independent manner. **a**–**d** NCI-H3122 cells were transfected with the indicated siRNAs for 48 h and further treated with or without TGFβ (1 ng/mL) for 30 min prior to western blot analysis. **e**, **f** A549 cells were treated with TGFβ (1 ng/mL), crizotinib (the indicated concentrations or 10 μM), or both for 30 min or the indicated times prior to western blot analysis. **g** A549 cells were treated with TGFβ (5 ng/mL), crizotinib (1 or 10 μM), or both for 24 h prior to western blot analysis. **h** Calu-1 cells were transfected with a pGL2-3TP-luciferase plasmid for 24 h and further incubated with TGFβ (1 ng/mL), crizotinib (the indicated concentrations), or both for 24 h. Luciferase activity was expressed as a relative value compared to that of the untreated cells, which was set to 100%. The data are expressed as the mean ± SEM (*n* = 6). **p < 0.01 and ***p < 0.005. **i** Calu-1 cells were treated with TGFβ (1 ng/mL), crizotinib (the indicated concentrations), or both for 30 min prior to western blot analysis. **j** Calu-1 cells were treated with TGFβ (5 ng/mL), crizotinib (1 or 10 μM), or both for 24 h prior to western blot analysis. **k**–**n** A549 cells were treated with TGFβ (1 ng/mL), ALK-specific inhibitors (1 or 10 M), MET-specific inhibitors (1 or 10 μM), or two of them for 30 min prior to western blot analysis.

We first questioned whether crizotinib inhibits TGFβ signaling by an on-target mechanism (e.g., through an ALK-, MET-, RON-, or ROS1-dependent mechanism). When siRNAs against ALK, MET, or RON were employed in the experiments, these siRNAs did not interfere with TGFβ-mediated luciferase activity in NCI-H3122 cells (Supplementary Fig. 4a–c). In contrast, siTβRI, as a positive control, noticeably inhibited TGFβ signaling (Supplementary Fig. 4d). Western blot analysis also showed that siALK, siMET, or siRON did not affect TGFβ-induced Smad3 phosphorylation in NCI-H3122 cells compared to siTβRI (Fig. 2a–d and Supplementary Fig. 4e–h). Since our RT-PCR analysis found that ROS1 is not detectable in NCI-H3122 cells as previously described in refs. ^32,33^, we excluded the involvement of ROS1 in crizotinib-mediated inhibition of TGFβ signaling. Therefore, our results demonstrate that crizotinib attenuates TGFβ signaling through an ALK/MET/RON/ROS1-independent mechanism.

Then, we further examined the effect of crizotinib on TGFβ signaling in EML4-ALK-negative A549 and Calu-1 cells. Similar to the results obtained from NCI-H3122 cells (Fig. 1a–c), crizotinib inhibited TGFβ-mediated luciferase activity and Smad3 phosphorylation in a dose-dependent manner (Supplementary Figs. 5,6 and Fig. 2e, h, i). Crizotinib also inhibited TGFβ-induced Smad3 phosphorylation in EML4-ALK-positive (H2228 and SNU2535) and EML4-ALK-negative (H1975, Calu-3, and PC-9) NSCLC cells (Supplementary Fig. 7). The time course analysis also showed that crizotinib almost completely blocked TGFβ-induced Smad3 phosphorylation (Fig. 2f). Since TGFβ is known to induce EMT, which facilitates the migration and invasion of cancer cells^34,35^, we analyzed the expression levels of EMT marker proteins in A549 cells. TGFβ decreased the expression levels of E-cadherin and increased those of vimentin and fibronectin, and these effects of TGFβ were abolished by crizotinib (Fig. 2g, j). In addition, other ALK- or MET-specific inhibitors did not affect TGFβ-induced Smad3 phosphorylation (Fig. 2k–n) in A549 cells. Therefore, these results confirm that crizotinib inhibits TGFβ signaling through an off-target mechanism.

### Crizotinib directly inhibits TβRI kinase activity through competitive inhibition

**Fig. 3:**
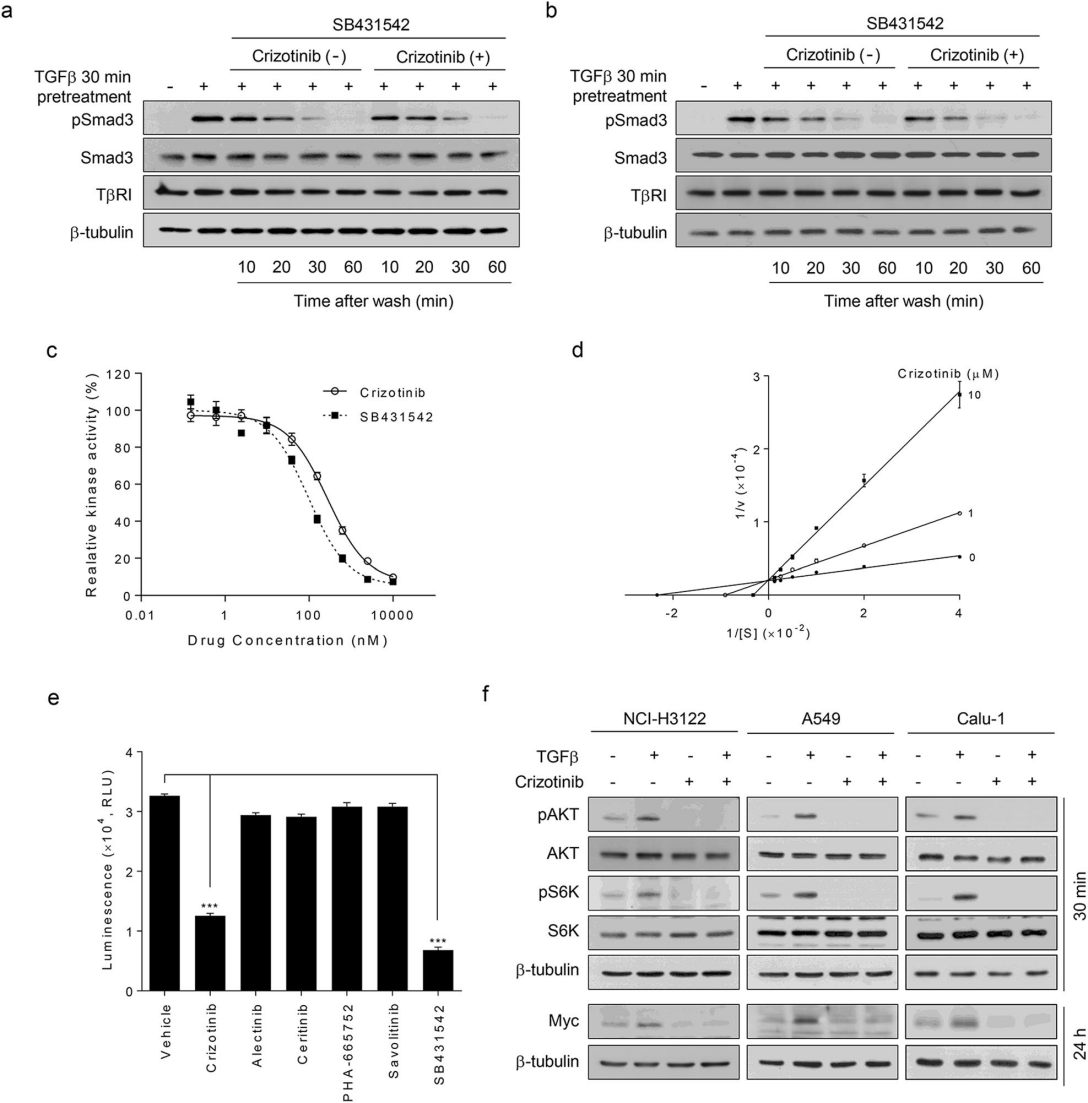
Crizotinib inhibits in vitro TβRI kinase activity. NCI-H3122 (**a**) and A549 (**b**) cells were pretreated with TGFβ (1 ng/mL) for 30 min, washed, and further incubated with or without crizotinib (10 μM) in the presence of SB431542 (10 μM) for the indicated times prior to western blot analysis. **c** The in vitro kinase activity of TβRI was measured in the presence of crizotinib or SB431542 (serially diluted in fourfold increments from 0.000153 to 10 μM) and expressed as luminescence signal values (relative light unit, RLU). The data were expressed as the mean ± SEM (*n* = 4). **d** Lineweaver–Burk plot analysis of 1/ν versus 1/[S]. The in vitro kinase activity of TβRI was measured at various concentrations of ATP (25, 50, 100, 200, 400, and 800 μM) in the presence of crizotinib (1 or 10 μM). The data were expressed as the mean ± SEM (*n* = 4). S, ATP. **e** Each indicated inhibitor at 10 μM was added to the enzyme reaction mixtures. The in vitro kinase activity of TβRI was expressed as luminescence signal values. The data were expressed as the mean ± SEM (*n* = 4). ***p < 0.005. **f** NCI-H3122, A549, and Calu-1 cells were treated with TGFβ (1 ng/mL), crizotinib (10 μM), or both for 30 min or 24 h prior to western blot analysis.

To determine the mechanism by which crizotinib inhibits TGFβ-induced Smad3 phosphorylation, we first assessed whether crizotinib stimulates the dephosphorylation of Smad3. After pretreatment with TGFβ for 30 min, the cells were washed and then treated with the TβRI kinase inhibitor SB431542 to prevent rephosphorylation of the dephosphorylated Smad3. Under this assay condition, crizotinib did not affect the phosphorylation levels of Smad3 in NCI-H3122 (Fig. 3a) and A549 cells (Fig. 3b). We also ascertained that crizotinib did not affect the expression level of TβRI (Fig. 3a, b). These results indicate that crizotinib inhibits the phosphorylation of Smad3 rather than stimulating its dephosphorylation or degradation of TβRI.

Based on these results (Fig. 3a, b), we investigated whether crizotinib directly acts on the kinase activity of TβRI. Molecular docking and amino acid sequence analyses provided clues to the inhibitory mechanism of crizotinib on TβRI; our model showed that crizotinib directly binds to the kinase domain of TβRI (Supplementary Figs. 8a–c, 9; see their legends for details). If our computational model is correct, crizotinib should directly inhibit the kinase activity of TβRI. To examine this assumption, we performed an in vitro ADP-Glo kinase activity assay using active recombinant TβRI and its substrate peptide. Crizotinib inhibited the in vitro kinase activity of TβRI, similar to the TβRI inhibitor SB431542, in a concentration-dependent manner (the IC_50_ values of crizotinib and SB431542 were 276.9 and 96.88 nM, respectively) (Fig. 3c). Therefore, these results demonstrate that crizotinib suppresses TGFβ signaling by directly inhibiting the kinase activity of TβRI.

Since molecular docking analysis also showed that crizotinib acts as an ATP-competitive inhibitor for TβRI (Supplementary Figs. 8, 9), we measured TβRI activity at various ATP concentrations in the presence of increasing concentrations of crizotinib. Lineweaver–Burk plot analysis showed that V_max_ was not changed, whereas K_m_ was increased (Fig. 3d), revealing that crizotinib behaves as an ATP-competitive inhibitor for TβRI. Altogether, our results demonstrate that crizotinib directly inhibits the kinase activity of TβRI in a competitive inhibition mode. We also found that other ALK- or MET-specific inhibitors did not affect the in vitro kinase activity of TβRI (Fig. 3e), confirming the specificity of crizotinib action on TβRI inhibition. If crizotinib directly inhibits TβRI activity, it should suppress noncanonical TGFβ signaling pathways, such as AKT, S6K, and Myc. Western blot analysis showed that crizotinib inhibited TGFβ-mediated AKT and S6K activation and Myc induction in NCI-H3122, A549, and Calu-1 cells (Fig. 3f). Altogether, our results demonstrate that crizotinib directly inhibits TβRI activity in a competitive inhibitory manner.

### Crizotinib suppresses TGFβ-induced cell migration and invasion

**Fig. 4:**
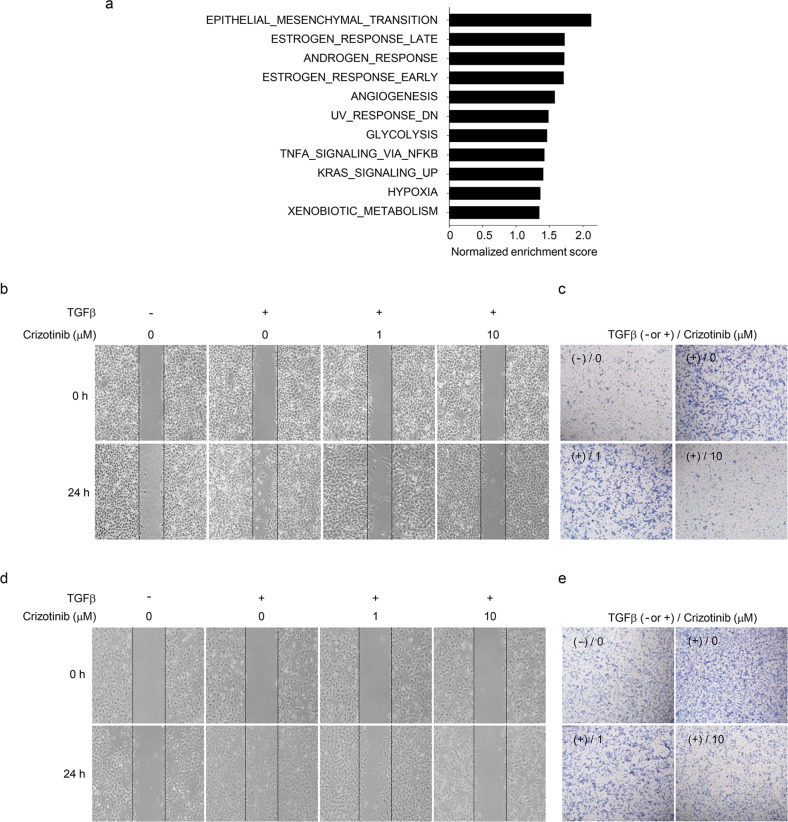
Crizotinib suppresses TGFβ-mediated cell migration and invasion. **a** Hallmark pathways affected by crizotinib in TGFβ-treated A549 cells. **b**, **d** Representative phase-contrast microscopic images (×40) of the scratch-wound assay. After scratching, A549 (**b**) and Calu-1 (**d**) cells were treated with TGFβ (5 ng/mL), crizotinib (1 or 10 μM), or both for 24 h. **c**, **e** Representative images (×40) of the transwell migration assay. A549 (**c**) and Calu-1 (**e**) cells were treated with TGFβ (5 ng/mL), crizotinib (1 or 10 μM), or both for 24 h. Cells that migrated to the lower chambers were stained with 0.2% crystal violet and photographed using a phase-contrast microscope.

TGFβ is known to regulate the migratory behaviors and invasion-metastasis cascade of cancer cells^30,34^. In addition, our own microarray experiments revealed that crizotinib significantly affected the expression of EMT signature genes in A549 cells (Supplementary Fig. 10 and Fig. 4a). To address the pathophysiological significance of our findings, we first examined whether crizotinib inhibits the migration of A549 cells treated with TGFβ, crizotinib, or both for 24 h. A scratch-wound assay showed that crizotinib decreased TGFβ-induced cell migration (Fig. 4b and Supplementary Fig. 11a). In addition, a transwell migration assay showed that crizotinib inhibited TGFβ-mediated invasion of A549 cells (Fig. 4c and Supplementary Fig. 11b). Similarly, crizotinib suppressed TGFβ-induced cell migration (Fig. 4d and Supplementary Fig. 11c) and invasion (Fig. 4e and Supplementary Fig. 11d) of Calu-1 cells. These results demonstrate that crizotinib potently inhibits TGFβ-induced migration and invasion of cancer cells.

**Fig. 5:**
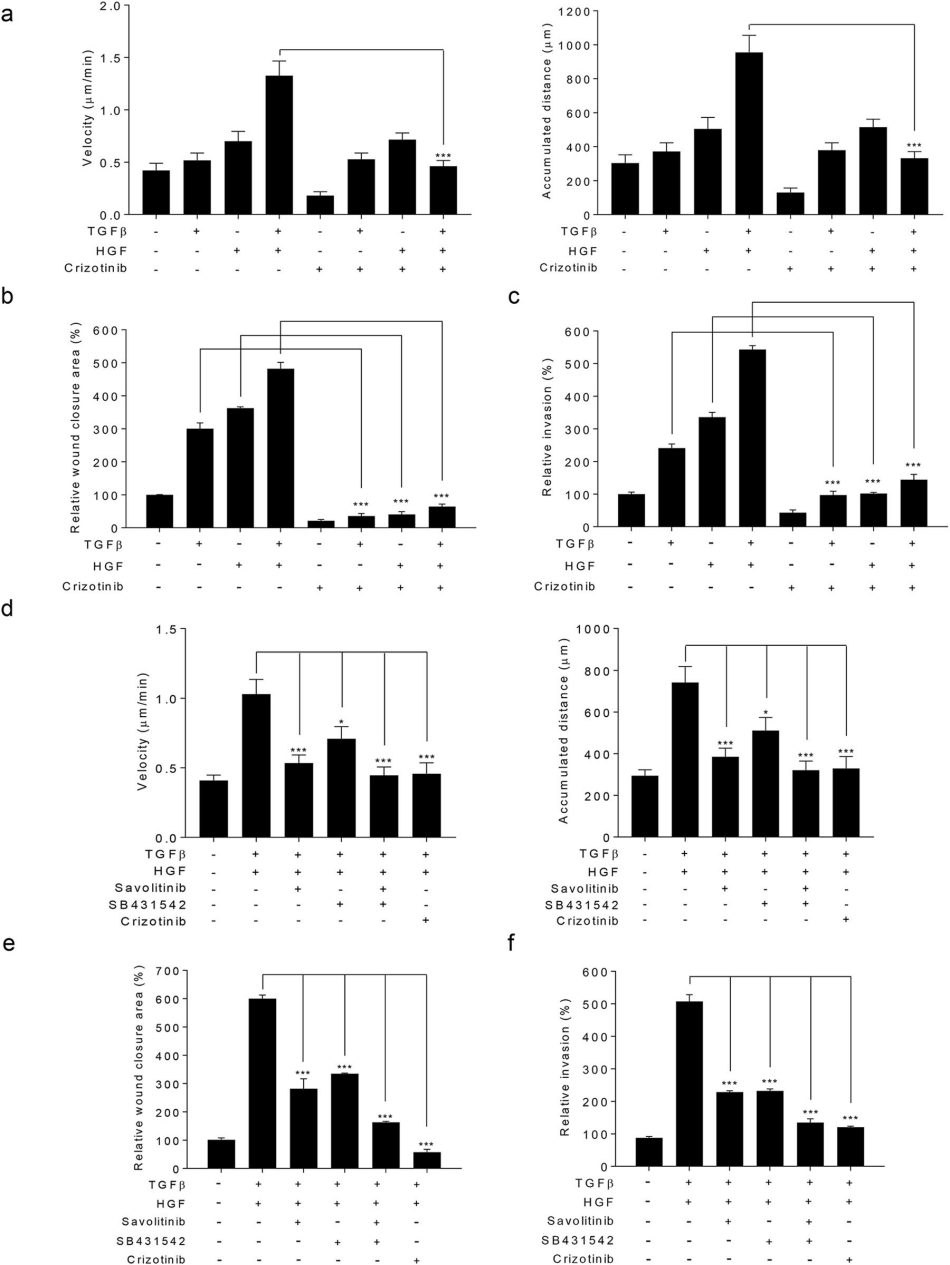
Crizotinib simultaneously suppresses TGFβ- and HGF-induced cell behaviors. **a**, **d** A549 cells were treated with TGFβ (5 ng/mL), HGF (50 ng/mL), crizotinib (10 μM), savolitinib (10 μM), SB431542 (10 μM), or some of them for 12 h. The velocity and accumulated distance of migrated cells were quantified from 2 min interval images using the BioStation time-lapse system and ImageJ software. The data were expressed as the mean ± SEM (*n* = 15). *p < 0.05, ***p < 0.005. **b**, **e** After scratching, A549 cells were treated with TGFβ (5 ng/mL), HGF (50 ng/mL), crizotinib (10 μM), savolitinib (10 μM), SB431542 (10 μM), or some of them for 24 h. The wound closure area was expressed as a relative value compared to that of untreated cells, which was set to 100%. The data were expressed as the mean ± SEM (*n* = 10). ***p < 0.005. **c**, **f** A549 cells were treated with TGFβ (5 ng/mL), HGF (50 ng/mL), crizotinib (10 μM), savolitinib (10 μM), SB431542 (10 μM), or some of them for 24 h. A549 cells that invaded the lower chambers were counted under a phase-contrast microscope. Invasion is expressed as a relative value compared to that of untreated cells, which was set to 100%. The data are expressed as the mean ± SEM (*n* = 5). ***p < 0.005.

The TGFβ and MET signaling pathways participate in the development of cancer metastasis^6,7^. The present study found that crizotinib concomitantly inhibits the TGFβ and MET signaling pathways (Fig. 1b, c). Based on these results, we investigated whether crizotinib suppresses cell migration and invasion by simultaneously inhibiting TGFβ and MET signaling in A549 cells. Cell tracking analysis showed that TGFβ, HGF (a ligand for MET), or both increased the velocity and accumulated distance of cell migration, which was reverted to basal levels by crizotinib (Fig. 5a and Supplementary Fig. 12a, b). In addition, scratch-wound and transwell migration assays showed that TGFβ, HGF, or both markedly increased cancer cell migration and invasion, and these effects were abolished by crizotinib (Fig. 5b, c and Supplementary Fig. 13a, b). These results demonstrate that crizotinib attenuates cell migration and invasion by concurrent inhibition of TGFβ and MET signaling.

To assess the potential benefit of simultaneous inhibition of two independent metastasis-related signaling pathways, we examined the effect of savolitinib, SB431542, and crizotinib on the migration and invasion of A549 cells in the presence of both TGFβ and HGF. As presented in Fig. 5d–f (also Supplementary Figs. 12c, d, 13c, d), combined treatment with savolitinib and SB431542 potently inhibited cell migration and invasion, which is comparable to crizotinib. In contrast, both savolitinib and SB431542 partially suppressed these cell behaviors. These results show that crizotinib acts as a useful multitarget antimetastatic agent for the treatment of advanced NSCLC.

### Crizotinib exerts antimetastatic activity in vivo

**Fig. 6:**
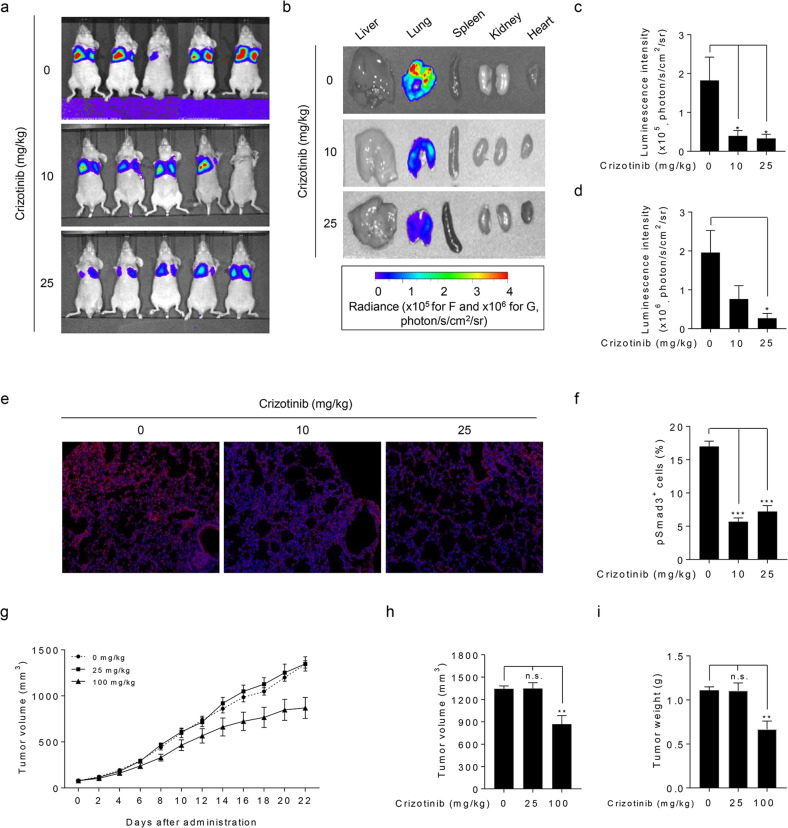
Crizotinib exerts antimetastatic activity in vivo. **a** Representative in vivo bioluminescence images were obtained 10 min after intraperitoneal injection of D-Luciferin in mice treated with crizotinib (0, 10, or 25 mg/kg) (see also Supplementary Fig. 14a). The radiance unit of photon/s/cm^2^/sr is the number of photons per second that leave a square centimeter of tissue and radiate into a solid angle of one steradian (sr). **b** Ex vivo bioluminescence images were measured from various isolated organs (see also Supplemental Fig. 14b). **c**, **d** Bioluminescence intensity was quantified for each mouse (**a**) or isolated lung tissue (**b**), and the mean value was calculated for each cohort. The data were expressed as the mean ± SEM (*n* = 5). *p < 0.01. **e**, **f** Phospho-Smad3-positive cells were assessed using immunofluorescence microscopic analysis in metastatic tumor tissues. Representative images of immunofluorescence staining (**e**) and quantification of the percentage of phospho-Smad3-positive cells (**f**). The data were expressed as the mean ± SEM (*n* = 160–424 fields from five tissues for each group). ***p < 0.005. **g**–**i** Tumor volumes were recorded twice (every other day) for 22 days. **g** The figures show the mean ± SEM (*n* = 5). **h**, **i** At 22 days after xenograft implantation, the mice were sacrificed to determine tumor volume (**h**) and weight (**i**). The figures show the mean ± SEM (*n* = 5). **p < 0.01. n.s. not significant.

We then investigated whether crizotinib suppresses tumor metastasis in vivo using highly metastatic Calu-1 NSCLC cells, which readily colonize the lungs following intravenous injection^28^. In vivo bioluminescence imaging analysis using luciferase-expressing Calu-1 (Calu-1-Luc) cells showed that luciferase activity was observed mainly in lung tissues compared to nonlung tissues (liver, spleen, kidney, and heart) in the tail-vein injection model of metastasis (Fig. 6a, b). Crizotinib markedly reduced luminescence signals from a lung metastasis of Calu-1-Luc cells (Fig. 6a, c, and Supplementary Fig. 14a). Ex vivo luminescence signals were observed mainly in isolated lung organs and were diminished by crizotinib (Fig. 6b, d and Supplementary Fig. 14b). In addition, immunofluorescence staining analysis showed that crizotinib decreased the number of phospho-Smad3-positive cells in metastatic cancer tissues (Fig. 6e, f). Tumor xenograft experiments showed that crizotinib at a dose of 25 mg/kg did not affect tumor growth compared to the 100 mg/kg dose (Fig. 6g–i), suggesting that the antimetastatic activity of crizotinib is independent of its antiproliferative activity. These results indicate that crizotinib suppresses metastatic colonization of circulating tumor cells without affecting tumor growth. These results demonstrate that crizotinib exerts antimetastatic activity in vivo, suggesting that crizotinib has a potential benefit for the prevention or inhibition of initial or recurrent metastasis and for the treatment of established metastatic NSCLC.

## Discussion

Crizotinib is a clinically approved agent for the treatment of patients with NSCLC harboring EML4-ALK or ROS1 rearrangement^13–15,17^. However, the mechanism of the anticancer action of crizotinib is still largely unknown. In this study, we identified a novel molecular mechanism by which crizotinib suppresses the metastatic capacity of NSCLC cells. Our main findings are as follows: (1) crizotinib blocks TGFβ-induced Smad activation in NSCLC cells via an ALK/MET/RON/ROS1-independent mechanism; (2) crizotinib directly inhibits TβRI kinase activity in a competitive inhibitory manner; (3) crizotinib suppresses TGFβ- and HGF-induced cell migration and invasion in NSCLC cells; and (4) crizotinib exerts antimetastatic activity in NSCLC cells in vivo without affecting cell growth.

Based on previous findings that MET is involved in the development of tumor metastasis^19–21,23^ and because crizotinib was originally developed as a MET inhibitor^16^, the possibility was raised that crizotinib can suppress tumor metastasis. However, crizotinib exerts antimetastatic activity in NSCLC cells. Our bioinformatic analysis provided a clue to the novel off-target mechanism of action of crizotinib (Table 1 and Supplementary Fig. 2). Based on our hypothesis- and data-driven inference and subsequent experiments, we found that crizotinib potently suppressed cell migration and invasion by simultaneous inhibition of at least two independent prometastatic signaling pathways involving the HGF-MET and TGFβ-TβRI pathways (Fig. 5), which contributes to its antimetastatic activity (Fig. 6). Therefore, these results deepen our understanding of the mechanism of the anticancer action of crizotinib and provide insight into the clinical usefulness of crizotinib in the treatment of metastatic cancer.

We found that TGFβ induces Smad3 phosphorylation without affecting ALK or MET phosphorylation (Fig. 1b, c), indicating that TGFβ signaling does not regulate the activity of ALK or MET signaling. Conversely, inhibition of ALK or MET using siRNAs or specific inhibitors did not affect TGFβ-induced Smad3 phosphorylation (Fig. 2k, l and Supplementary Fig. 4e, f), indicating that ALK or MET signaling does not lead to deregulated TGFβ signaling. Therefore, our results demonstrate that these oncogenic signaling pathways are independent of each other and independently contribute to tumor metastasis, suggesting that simultaneous inhibition of multiple oncogenic pathways is crucial for the prevention or inhibition of metastasis.

Our findings suggest that crizotinib-resistant metastatic NSCLC cells can develop cellular resilience mechanisms to resist molecular or cellular perturbation by inhibition of TGFβ signaling as well as ALK and MET, providing new insight into the mechanism of cancer drug resistance. In this regard, it is possible that the tumor cells acquiring resistance to crizotinib-induced TGFβ inhibition may rapidly result in highly metastatic cancer. Therefore, careful clinical observation is required to assess the potential association between the crizotinib dose and metastatic progression. However, a previous study showed that aberrant TGFβ signaling plays a crucial role in the acquisition of resistance to crizotinib in ALK-rearranged NSCLC cells in response to chronic, repeated exposure to crizotinib^36^, suggesting the presence of a latent association between TGFβ and ALK signaling with respect to a complex mechanism of cancer drug resistance.

We showed that crizotinib inhibited TGFβ-induced EMT, cell migration, and invasion and attenuated metastatic colonization in NSCLC cells. Previous clinical studies have shown that crizotinib is beneficial for patients with established metastatic NSCLC^22^. Therefore, our results suggest that crizotinib may be useful for the prevention or inhibition of initial or recurrent metastasis and the treatment of established metastatic tumors. Since TGFβ plays a crucial role in immune evasion, angiogenesis, and metastatic niche establishment^2,6^, crizotinib can suppress tumor metastasis by targeting the tumor microenvironment (i.e., noncell autonomous mechanism), suggesting that crizotinib exerts multilayered, multitargeted antimetastatic properties. This phenomenon may explain the effectiveness of crizotinib in inhibiting cancer metastasis in vivo, although it exerted antimetastatic activity in our in vitro model at relatively high concentrations. In addition, our results suggest that crizotinib can be exploited as a valuable chemical probe for dissecting metastatic signaling networks in NSCLC, indicating that crizotinib can be used as a lead compound for structure-based optimization studies to maximize antimetastatic therapeutic efficacy.

The present study demonstrates that crizotinib attenuates cancer metastasis by ALK/MET/RON/ROS1-independent inhibition of TGFβ signaling in NSCLC cells. Our results will contribute to enhancing the understanding of the molecular mechanism underlying the anticancer actions of crizotinib and providing further insight into its clinical usefulness.

## Supplementary information

Supplementary information
