# Supplementary material for: Crizotinib attenuates cancer metastasis by inhibiting TGFβ signaling in non-small cell lung cancer cells
Source: Exp Mol Med. 2022 Aug 23;54(8):1225–35. doi: 10.1038/s12276-022-00835-8 (PMC9440021; doi:10.1038/s12276-022-00835-8)

Supplemental Figure Legends

Supplementary Fig. 1 Distinctive changes in gene expression in GSE89127 and GSE31210. (a, b) Hierarchical clustering illustrates a large quantity of DEGs in GSE89127 (NCI-H3122 cells treated with crizotinib) or GSE31210 (patients with NSCLC harboring EML4-ALK) datasets. The color indicator represents log_2 transformation. (c, d) PCA plots shows distinct clusters in GSE89127 and GSE31210 datasets. (e, f) Limma analysis identified 6,019 DEGs (2,367 downregulation and 3,652 upregulation) from GSE89127 dataset and 7,827 DEGs (3,598 downregulation and 4,229 upregulation) from GSE31210 dataset. Of these DEGs, 1,697 DEGs (868 + 829) were found to exhibit anti-similarity (or inverse correlation) between two datasets.

Supplementary Fig. 2 Crizotinib suppresses the EMT signature. The heatmap shows the expression levels of genes involved in the EMT signature, displaying anti-similarity (or inverse correlation) between GSE89127 and GSE31210 datasets. We examined anti-similarity (inverse correlation) of gene expression signatures between GSE89127 and GSE31210 datasets, based on REACTOME and Gene Ontology Biological Process data from Molecular Signature Database.

Supplementary Fig. 3 Crizotinib inhibits TGFβ-mediated luciferase activity. NCI-H3122 cells were transfected with pGL2-3TP-luciferase plasmid for 24 h and further incubated with TGFβ (the indicated concentrations), crizotinib (the indicated concentrations), or both for 24 h. Luciferase activity was expressed as a relative value compared to that of the untreated cells

which was set to 100%. The data were expressed as the mean ± SEM (n=6). $^{**}p < 0.01$, $^{***}p < 0.005$.

Supplementary Fig. 4 ALK, MET, or RON does not affect TGFβ signaling pathway. (a-d) NCI-H3122 cells were transfected with the indicated siRNAs for 24 h, then transfected with pGL2-3TP-luciferase plasmid for 24 h, and further incubated with TGFβ (1 ng/mL) for 24 h. Luciferase activity was expressed as a relative value compared to that of the untreated cells which was set to 100%. The data were expressed as the mean ± SEM (n=6). $^{***}p < 0.005$. n.s., not significant. (e-h) NCI-H3122 cells were transfected with the indicated siRNAs for 48 h, and further treated with or without TGFβ (1 ng/mL) for 30 min prior to western blot analysis. The sequence of each siRNA is shown in Materials and Methods.

Supplementary Fig. 5 The comparison of protein expression or reactivity in NCI-H3122, A549, and Calu-1 cells. (a) The expression levels of phospho-ALK, ALK, phospho-MET, and MET in untreated cells. (b) The expression levels of phospho-Smad3, Smad3, and TβRI in TGFβ-treated cells. TGFβ (1 ng/mL) was treated for 30 min prior to western blot analysis.

Supplementary Fig. 6 Crizotinib inhibits TGFβ-mediated luciferase activity. A549 cells were transfected with pGL2-3TP-luciferase plasmid for 24 h and further incubated with TGFβ (1 ng/mL), crizotinib (the indicated concentrations), or both for 24 h. Luciferase activity was expressed as a relative value compared to that of the untreated cells which was set to 100%.

The data were expressed as the mean ± SEM (n=6). $^{*}p < 0.05$ and $^{***}p < 0.005$.

Supplementary Fig. 7. The comparison of protein expression or reactivity in H2228, SNU2535, H1975, Calu-3, and PC-9 cells. (a) These NSCLC cells were treated with TGFβ (1 ng/mL), crizotinib (10 μM), or both for 30 min prior to western blot analysis. (b) The expression levels of phospho-ALK, ALK, phospho-MET, and MET in untreated NSCLC cells.

Supplementary Fig. 8 Structural modeling of crizotinib-target complex. Structural coordinate of the kinase domain of TβRI was adopted from PDB code 3TZM, which was used to prepare ligand and receptor molecules. AutoDock parameter set- and distance-dependent dielectric functions were used in calculating van der Waals and electrostatic terms, respectively. Docking simulations were performed using Lamarckian genetic algorithm (LGA). Initial position, orientation, and torsions of ligand molecules were set randomly. All rotatable torsions were released during docking. For each docking experiment, 100 different runs of Lamarckian genetic search were performed and resulting docked ligand coordinates were clustered based on their relative mean standard deviation (r.m.s.d.) values. Most likely binding conformation and corresponding intramolecular interactions were analyzed and visualized using PyMol software. (a) Predicted binding of crizotinib to the kinase domain of TβRI or ALK. The bottom is a superposed image of crizotinib-binding cavity in TβRI and ALK. Our computational model showed that crizotinib directly binds to TβRI, exhibiting an estimated free binding energy of -5.94 kcal/mol (in ALK, -8.07 kcal/mol; in MET, -7.45 kcal/mol): Leu340, Lys337, and several polar amino acids (Arg215, Lys232, Glu245, Lys335, and Asp351) in the kinase domain of

TβRI participate in hydrogen bond formation, hydrophobic interaction, and dipole-dipole interactions with crizotinib, respectively. The superposed image showed that crizotinib is bound to TβRI and ALK at similar binding sites. However, the bond between crizotinib and TβRI showed a different direction to that between crizotinib and ALK. Since the space between the N- and C-lobes of the TβRI kinase domain is broad, crizotinib can bind in different directions. A similar case was also reported in a structural study of RON-crizotinib complex,[1] compared to crizotinib-ALK, -MET, or -ROS1 complexes. (b) Binding of crizotinib to TβRI, ALK, MET, ROS1, or RON. (c) Merged image.

[1]Cui JJ, Tran-Dube M, Shen H, Nambu M, Kung PP, Pairish M, Jia L, Meng J, Funk L, Botrous I et al. (2011) Structure based drug design of crizotinib (PF-02341066), a potent and selective dual inhibitor of mesenchymal-epithelial transition factor (c-MET) kinase and anaplastic lymphoma kinase (ALK). *J Med Chem.* 54: 6342-6363.

Supplementary Fig. 9 Sequence alignment of the ATP-binding pocket of ALK and TβRI. Sequences were aligned using 1093-1411 amino acids of ALK and 208-409 amino acids of TβRI. The amino acids with high consensus are indicated in red. The sequence alignment of TβRI and ALK showed that Gly1202, Asn1254, and Gly1269, crucial amino acids in ALK for binding to crizotinib,[1, 2] were conserved in the kinase domain of TβRI, suggesting that crizotinib can bind to TβRI in a similar manner with ALK. *Matching crucial amino acids between ALK and TβRI.

[1]Cui JJ, Tran-Dube M, Shen H, Nambu M, Kung PP, Pairish M, Jia L, Meng J, Funk L, Botrous I et al. (2011) Structure based drug design of crizotinib (PF-02341066), a potent and selective

dual inhibitor of mesenchymal-epithelial transition factor (c-MET) kinase and anaplastic lymphoma kinase (ALK). *J Med Chem.* 54: 6342-6363.

[2]Li J, Sun R, Wu Y, Song M, Li J, Yang Q, Chen X, Bao J, Zhao Qi (2017) L1198F Mutation Resensitizes Crizotinib to ALK by Altering the Conformation of Inhibitor and ATP Binding Sites. *Int J Mol Sci.* 18: 482-495.

Supplementary Fig. 10 Microarray experiment and bioinformatic analysis. DNA microarray experiments were performed total RNA from A549 cells following treatment with crizotinib (10 μM), TGFβ (1 ng/ml.), or both for 24 h. The microarray data are available through GEO database (GSE189047). Feature selection across four groups was performed using the Limma Bioconductor R package with a multiclass statistical problem type.[1] The Benjamini and Hochberg (BH) procedure was used for the adaptive control of the FDR in multiple testing.[2] The significant features were determined by the threshold FDR *q*-value 0.25. Internal clusters were validated by hierarchical clustering and principal component analysis using the selected features. (a) Hierarchical clustering analysis of GSE189047 illustrates the distinctive gene expression among the four experimental groups. (b) Principal Component Analysis (PCA) of GSE189047 shows distinctive clusters in the principal components. (c, d) Differentially expressed genes (DEGs) among experimental groups were presented as Venn diagrams. Among the 10,622 DEGs identified by Limma analysis, 254 (178 up-regulated and 76 down-regulated) genes were reversed by crizotinib in TGFβ-treated cells. The CSEA Hallmark analysis was performed using the 254 DEGs (see also Fig. 4a).

[1]Ritchie ME, Phipson B, Wu D, Hu Y, Law CW, Shi W, Smyth GK (2015) limma powers differential expression analyses for RNA-sequencing and microarray studies. *Nucleic acids Res.* 43, e47-e47.

[2]Benjamini, Y.; Hochberg, Y. (1995) Controlling the false discovery rate: a practical and powerful approach to multiple testing. *J. R. Sstat. Soc. B. Methodol.* 57: 289-300.

Supplementary Fig. 11 Crizotinib suppresses cell migration and invasion. (a, c) Quantification of scratch-wound assay results, which is related to Figs. 4b and d. After scratch, A549 (a) or Calu-1 (c) cells were treated with TGFβ (5 ng/mL), crizotinib (1 or 10 μM), or both for 24 h. Wound closure area was expressed as a relative value compared to that of untreated cells which was set to 100%. The data are expressed as the mean ± SEM (n=4-10). $^{***}p < 0.005$. (b, d) Quantification of transwell migration assay results, which is related to Figs. 4c and e. A549 (b) or Calu-1 (d) cells that invaded to the lower chambers were counted under a phase-contrast microscope. Invasion is expressed as a relative value compared to that of untreated cells which was set to 100%. The data are expressed as the mean ± SEM (n=4-5). $^{***}p < 0.005$.

Supplementary Fig. 12 Cell tracking using videomicroscopy in A549 cells. Cells were treated with TGFβ (5 ng/mL), HGF (50 ng/mL), crizotinib (10 μM), savolitinib (10 μM), SB431542 (10 μM), or some of them for 12 h (a and c) or 30 min (b and d). (a, c) Cell images were obtained in intervals of 2 min for 12 h under the indicated conditions. (b, d) The expression levels of phospho-Smad3, Smad3, phospho-MET, MET, and β-tubulin was determined by western blot analysis.

Supplementary Fig. 13 Representative images of scratch-wound and transwell assays in A549 cells. (a, c) Phase-contrast microscopic images (× 40) of scratch-wound assays, which is related to Figs. 5b and e. (b, d) Phase-contrast microscopic images (× 40) of transwell migration assays, which is related to Figs. 5c and f.

Supplementary Fig. 14 *In vivo* luminescence images of metastasis experiments. (a) *In vivo* bioluminescence images were obtained in mice treated with crizotinib (0, 10, 25 mg/kg). (b) Bioluminescence images were obtained from the various organs isolated after sacrifice.

Supplementary Fig. 15 Full scan images used in this study.

Supplementary Fig. 1

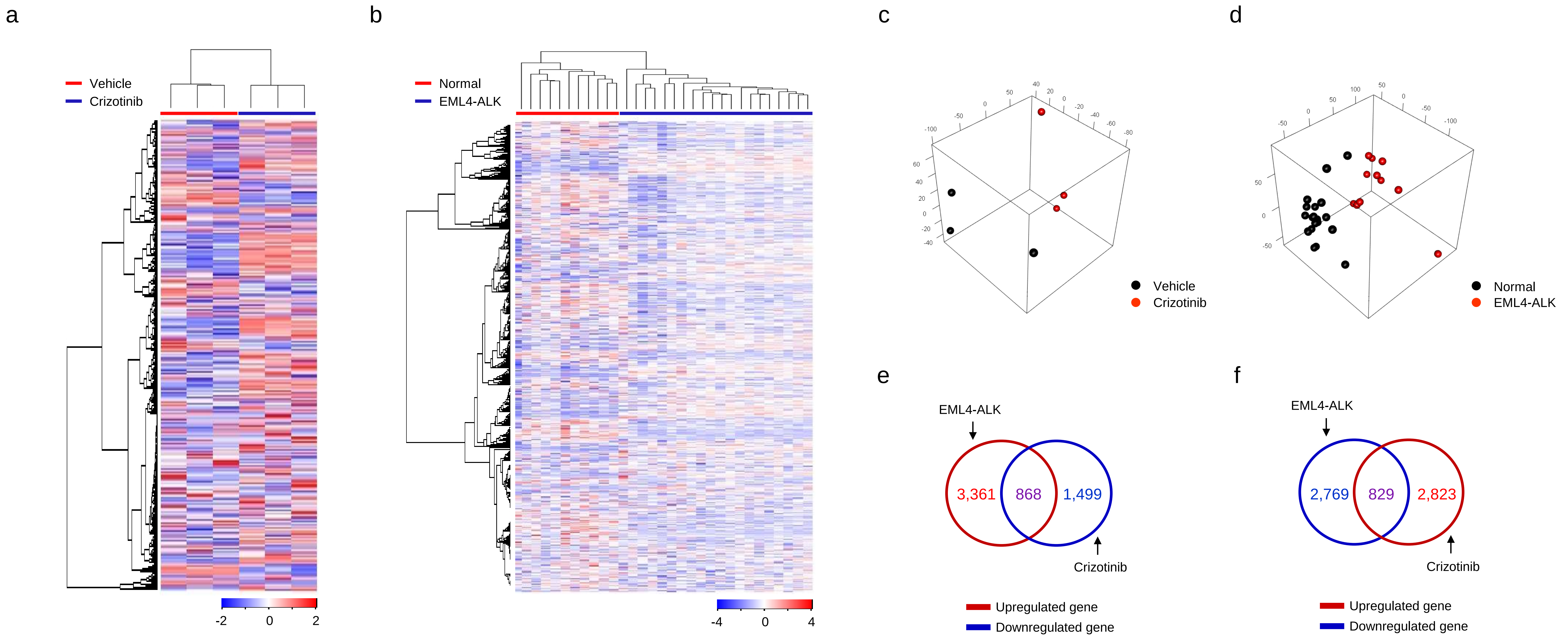

Supplementary Fig. 2

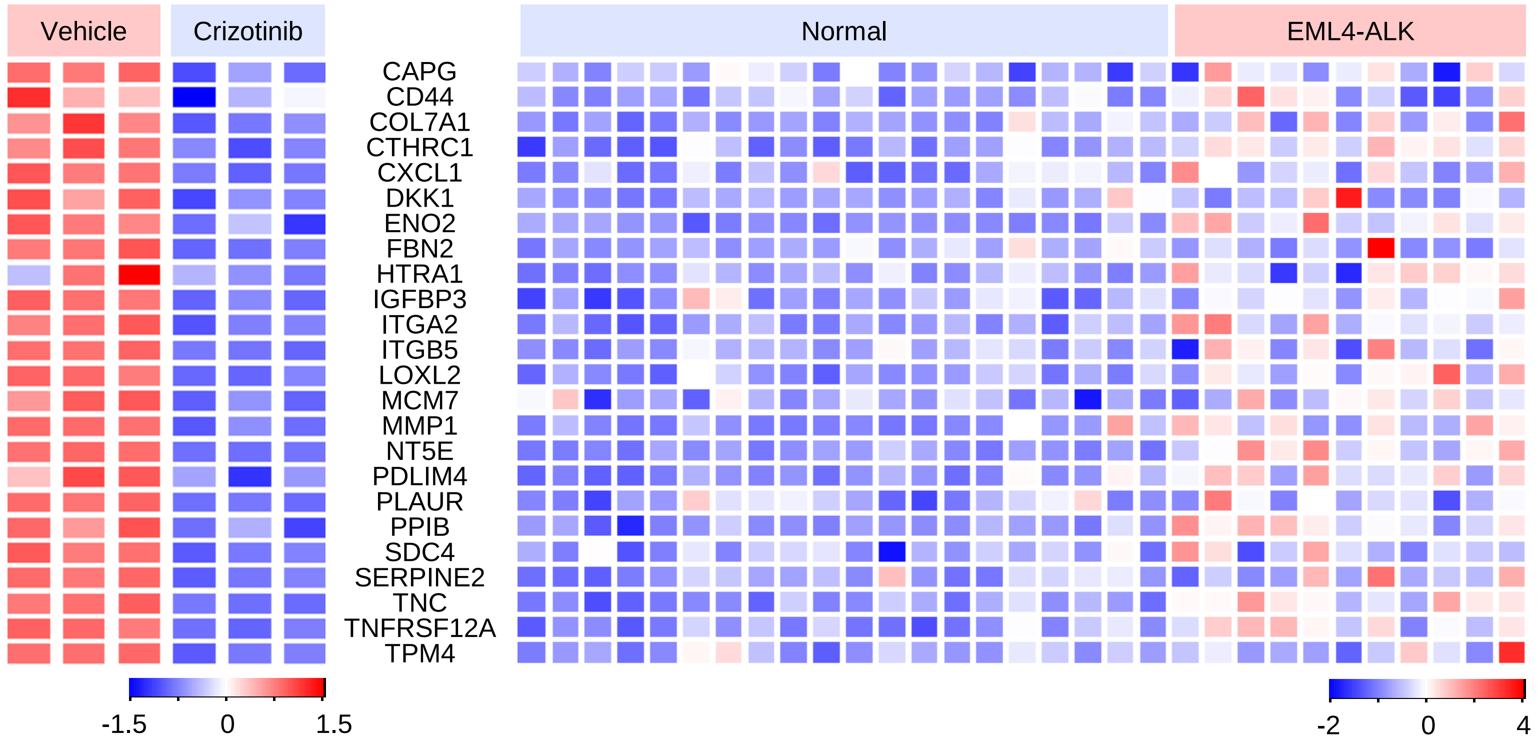

Supplementary Fig. 3

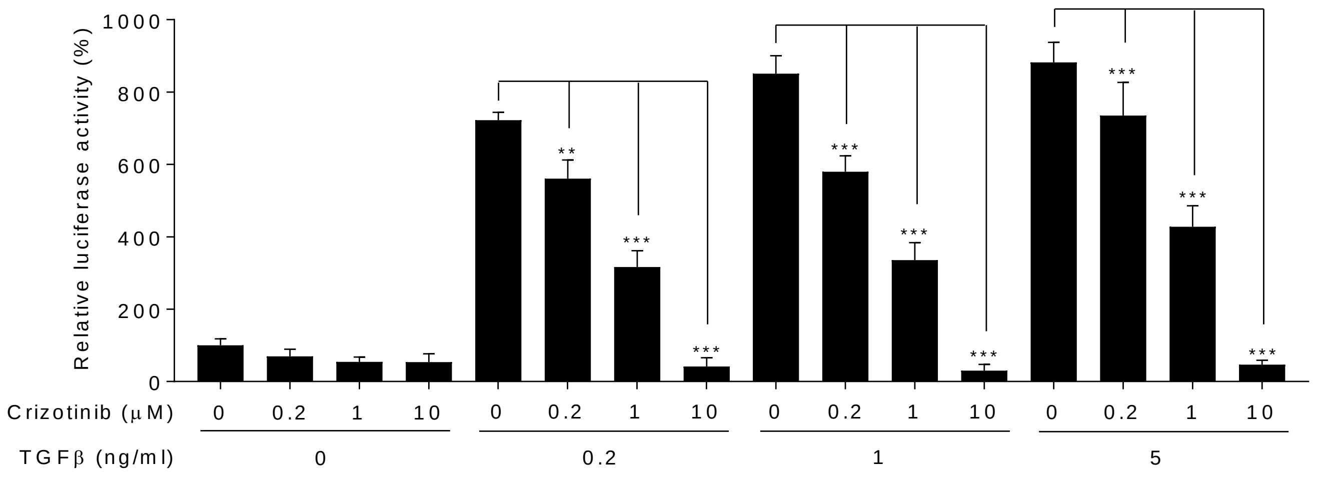

Supplementary Fig. 4

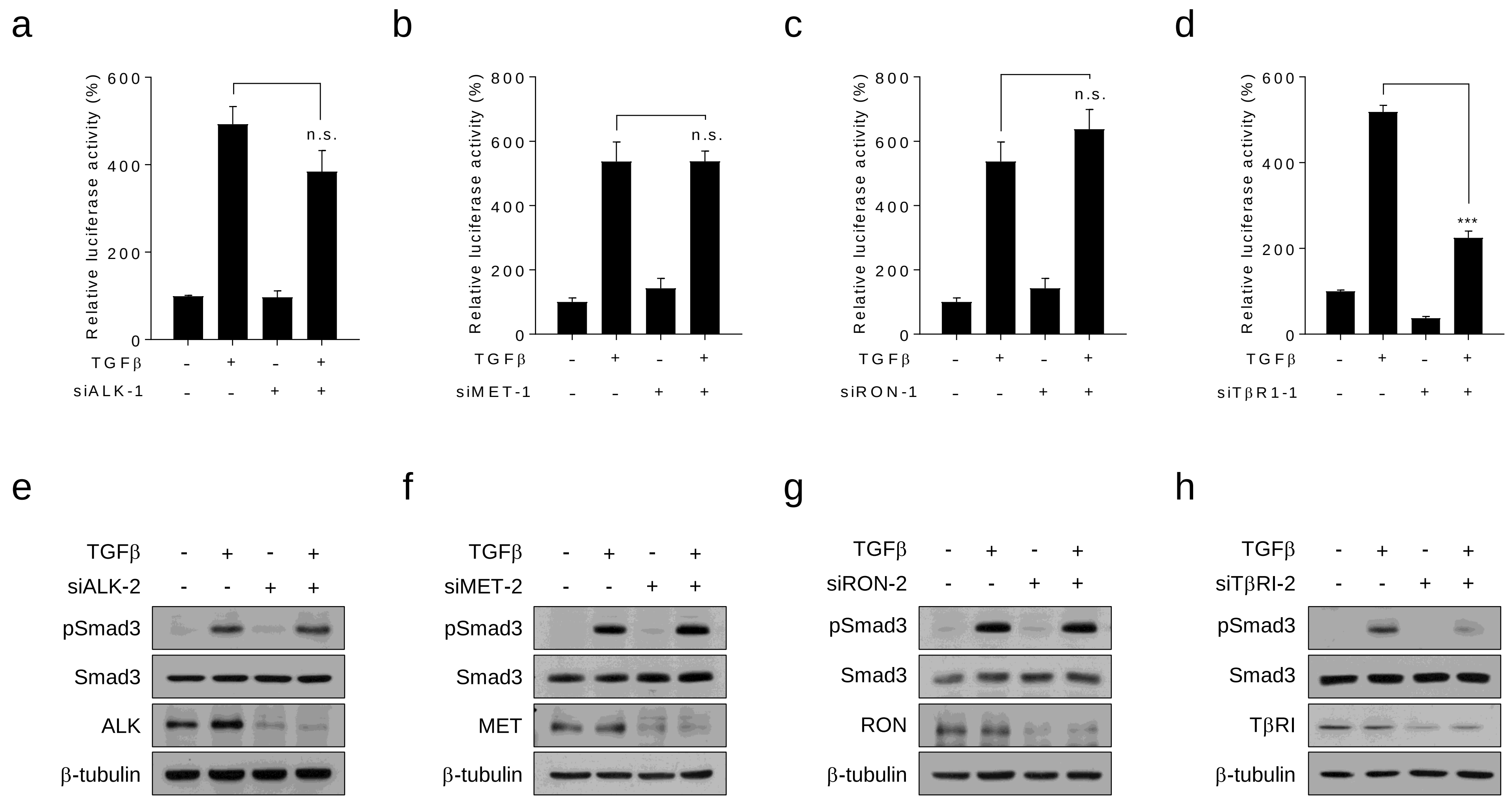

Supplementary Fig. 5

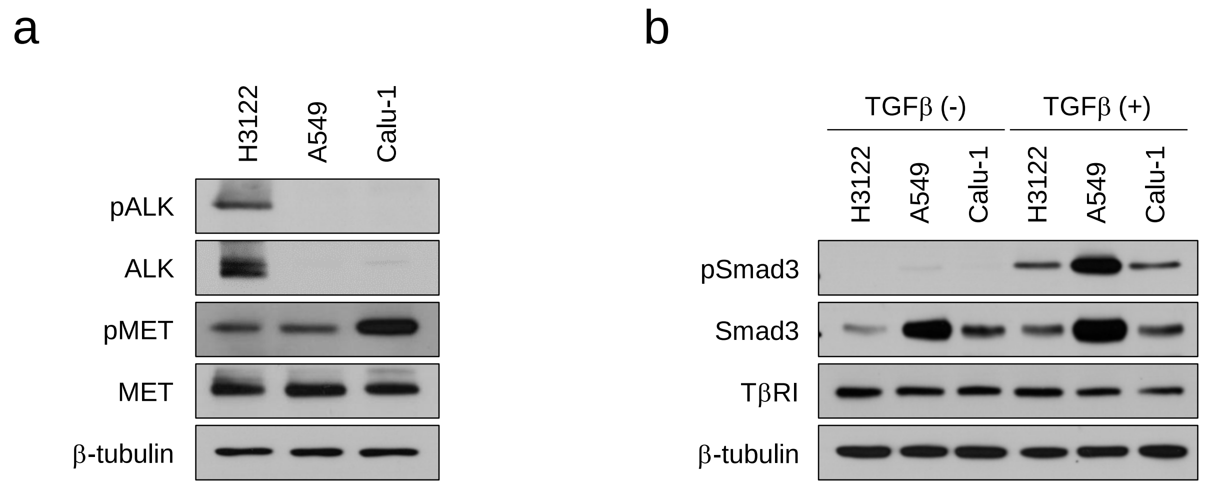

Supplementary Fig. 6

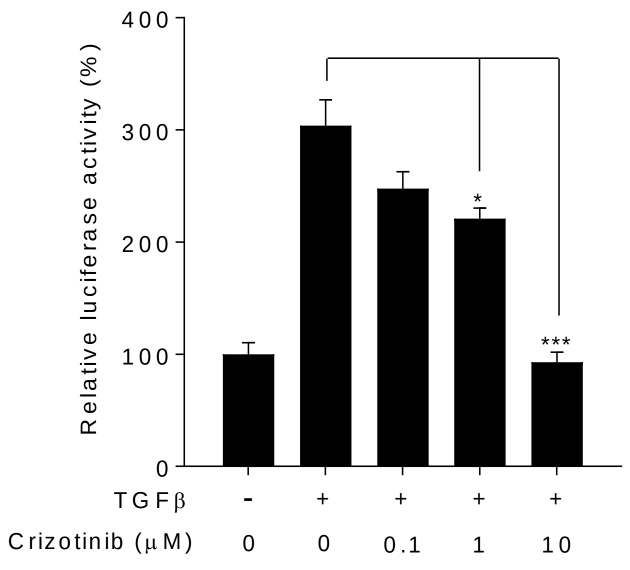

Supplementary Fig. 7

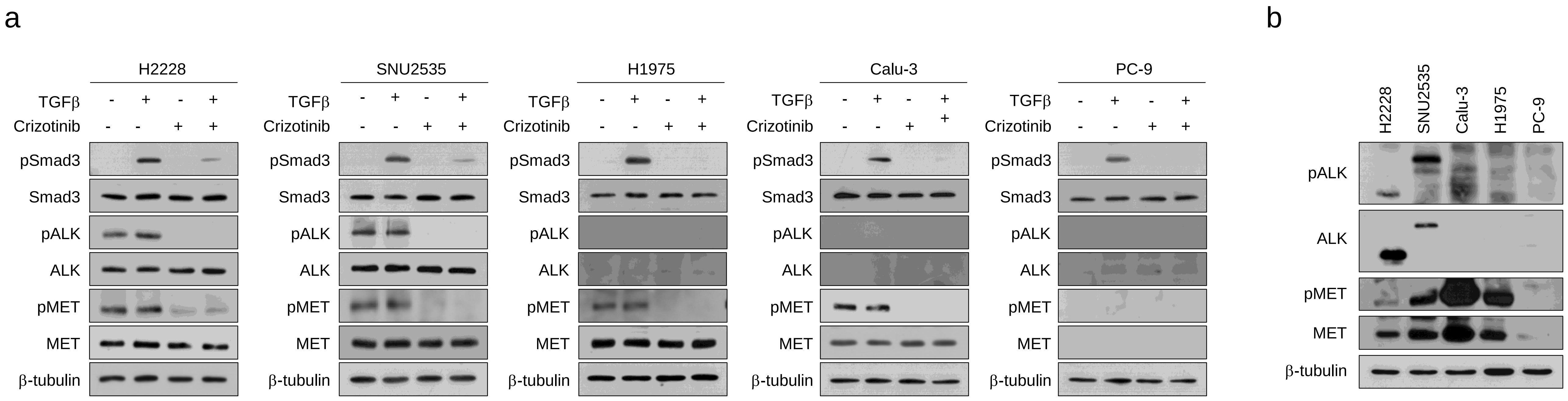

Supplementary Fig. 8

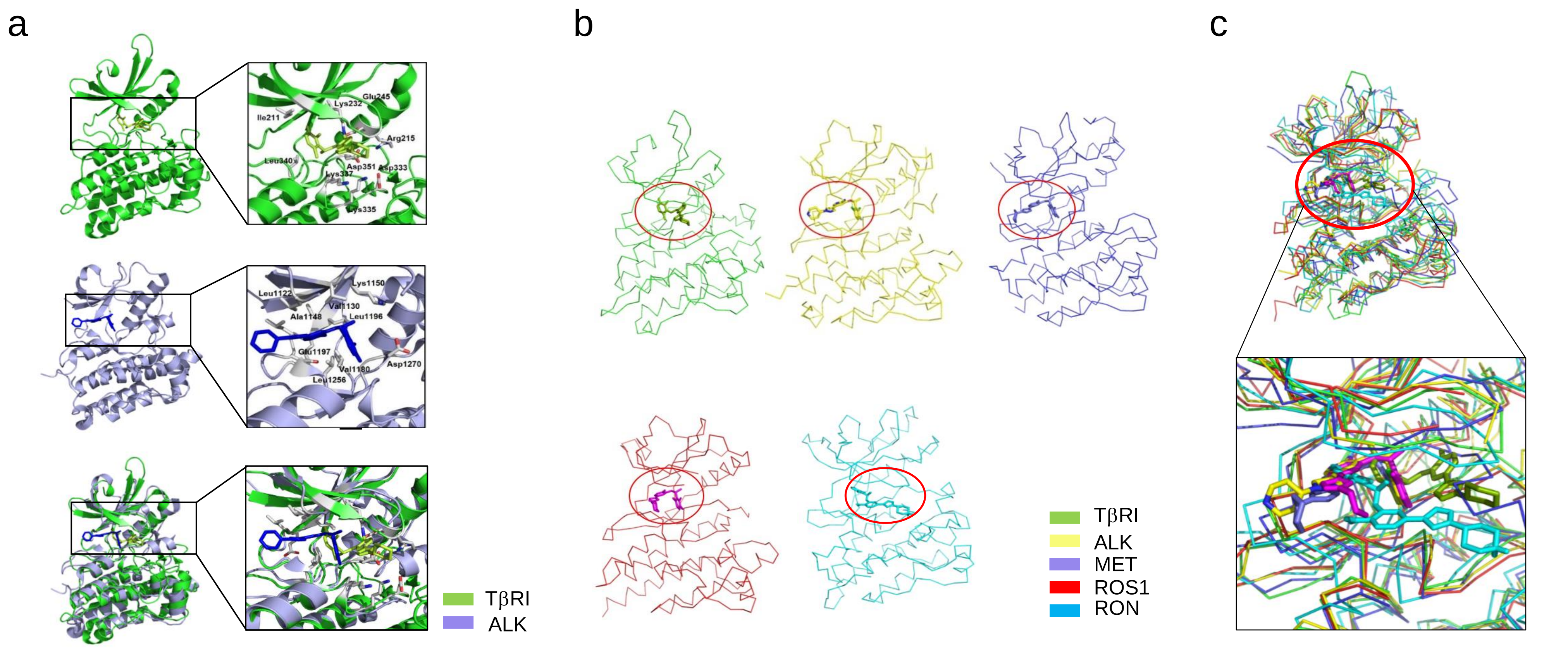

Supplementary Fig. 9

```
ALK  1093  ---------- ---------- ---------- GHGAFGEVYE GQVSGMPNDP SPLQVAVKTL
TβRI  208  ---------- ---------- ---------- GKGRFGEVWR GKWRGE---- ---EVAVK--
                                                                       *
ALK  1153  PEVCSEQDEL DFLMEALIIS K--FNHQNIV RCIGVSLQSL PRFILLELMA G----GDLKS
TβRI  233  --IFSSREER SWFREAEIYQ TVMLRHENIL GFIAADNKDN GTWTQLWLVS DYHEHGSLFD
                                                              *
ALK  1207  FLRETRPRPS QPSSLAMLDL LHVARDIACG CQYLEENHFI HRDIAARNCL LTCPGPGRVA
TβRI  291  YLNRYTVTVE GMIKLALSTA SGLAHLHMEI VGTQGKPAIA HRDLKSKNIL VKKNGTCCIA
             *
ALK  1267  KIGDFGMARD IYRASYYRKG GCAMLPVKWM PPEAFMEGI- ---FTS--KT DTWSFGVLLW
TβRI  351  DLG-LAVRHD SATDTIDIAP NHRVGTKRYM APEVLDDSIN MKHFESFKRA DIYAMGLVFW

ALK  1321  EIFSLGYMPY PSKS NQ-EVL EFVTSGGRMD PPKN--CPGP VYRIMTQCWQ HQPEDRPNFA
TβRI  410  EIARRCSIGG IHEDYQLPYY DLVPSDPSVE EMRKVVCEQK LRPNIPNRWQ SCEALRV-MA

ALK  1378  IILERIEYCT QDPDVINTAL PIE------- ----
TβRI  469  KIMRECWYAN GAARL--TAL RIK------- ----
```

Supplementary Fig. 10

a

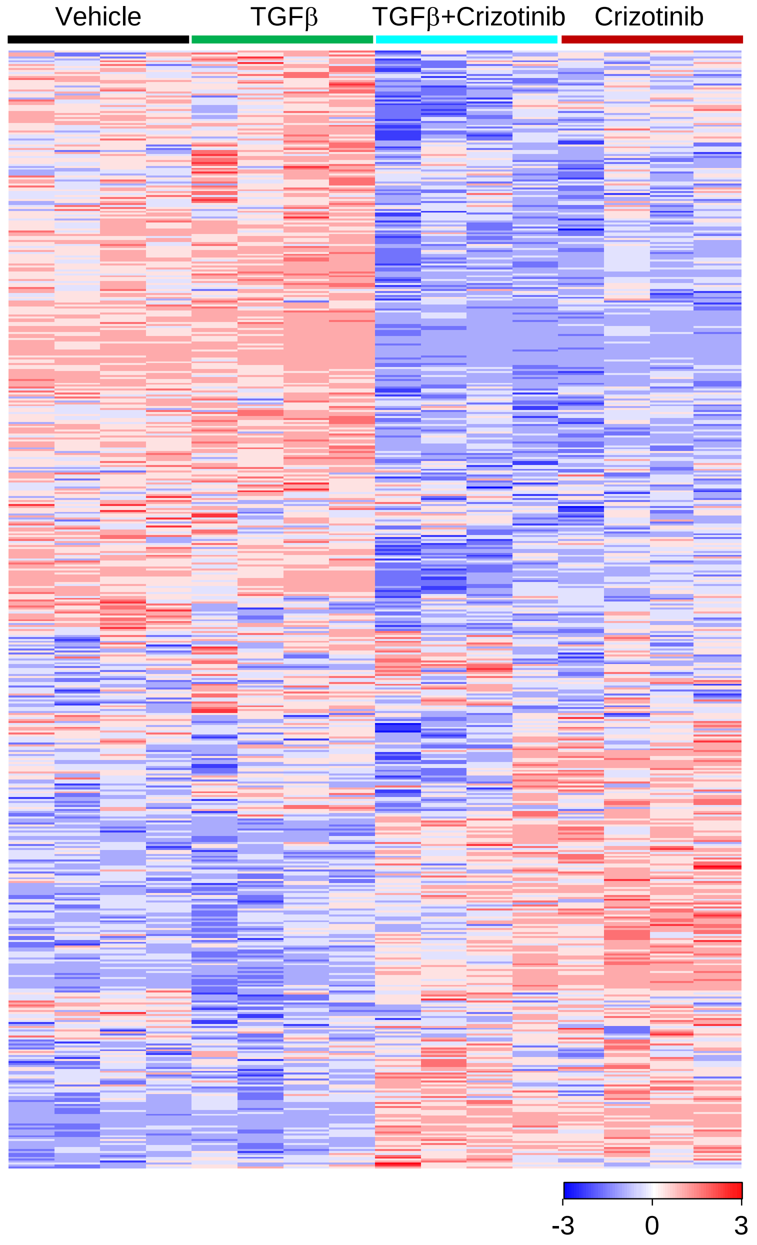

b

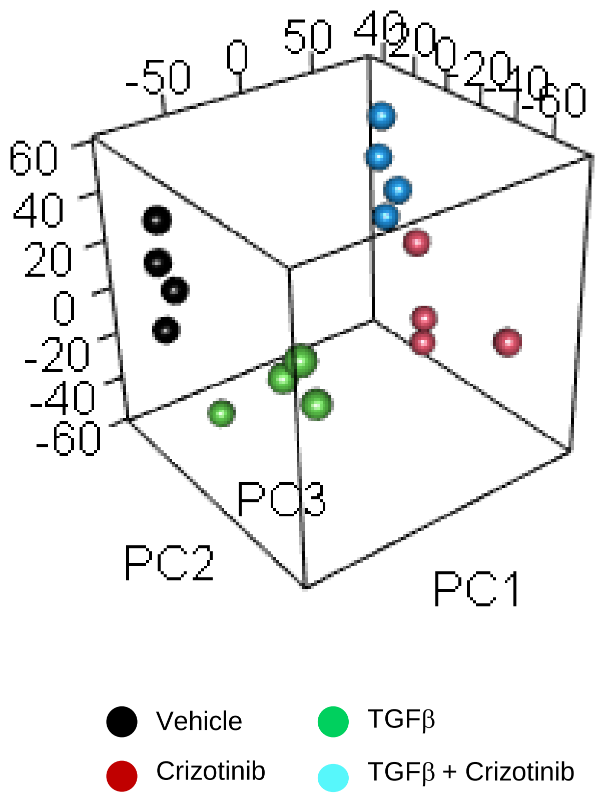

c

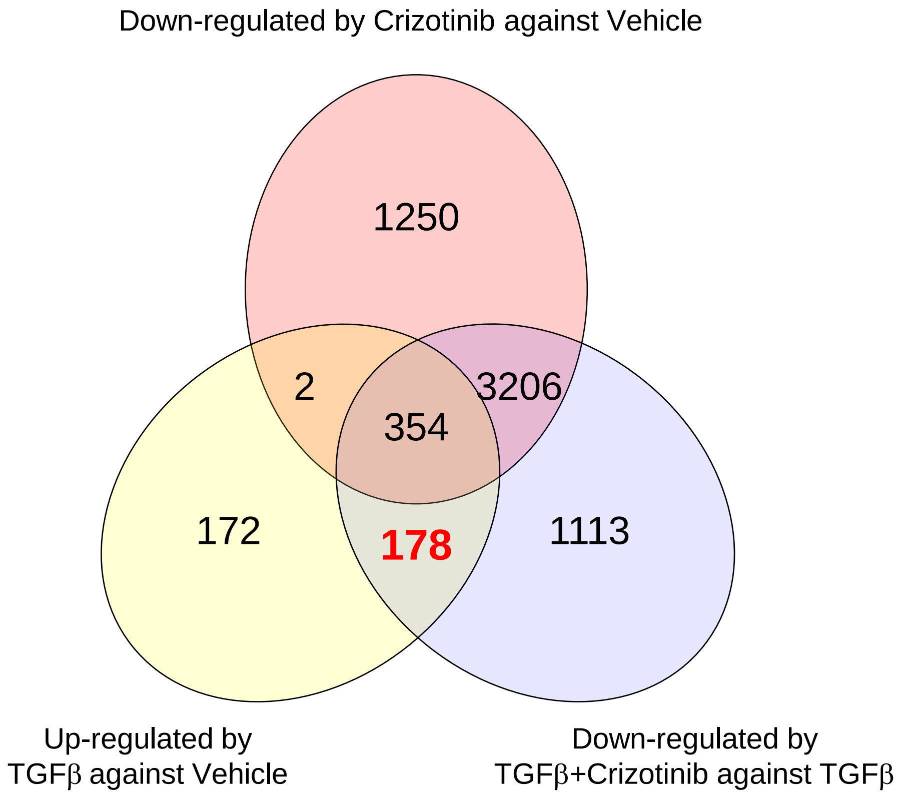

d

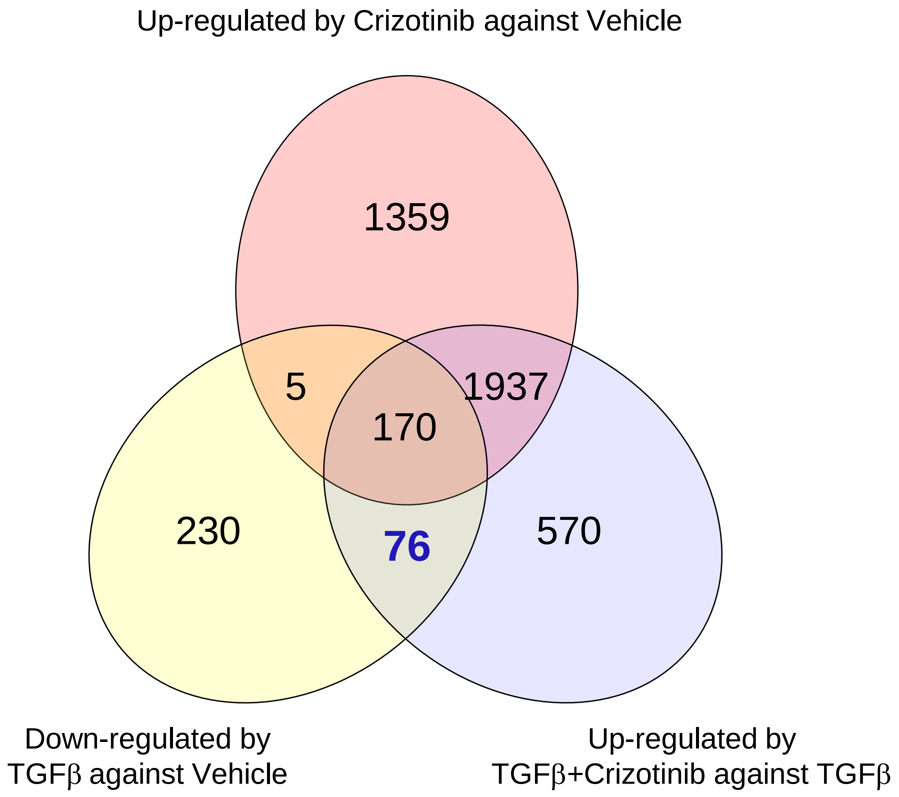

Supplementary Fig. 11

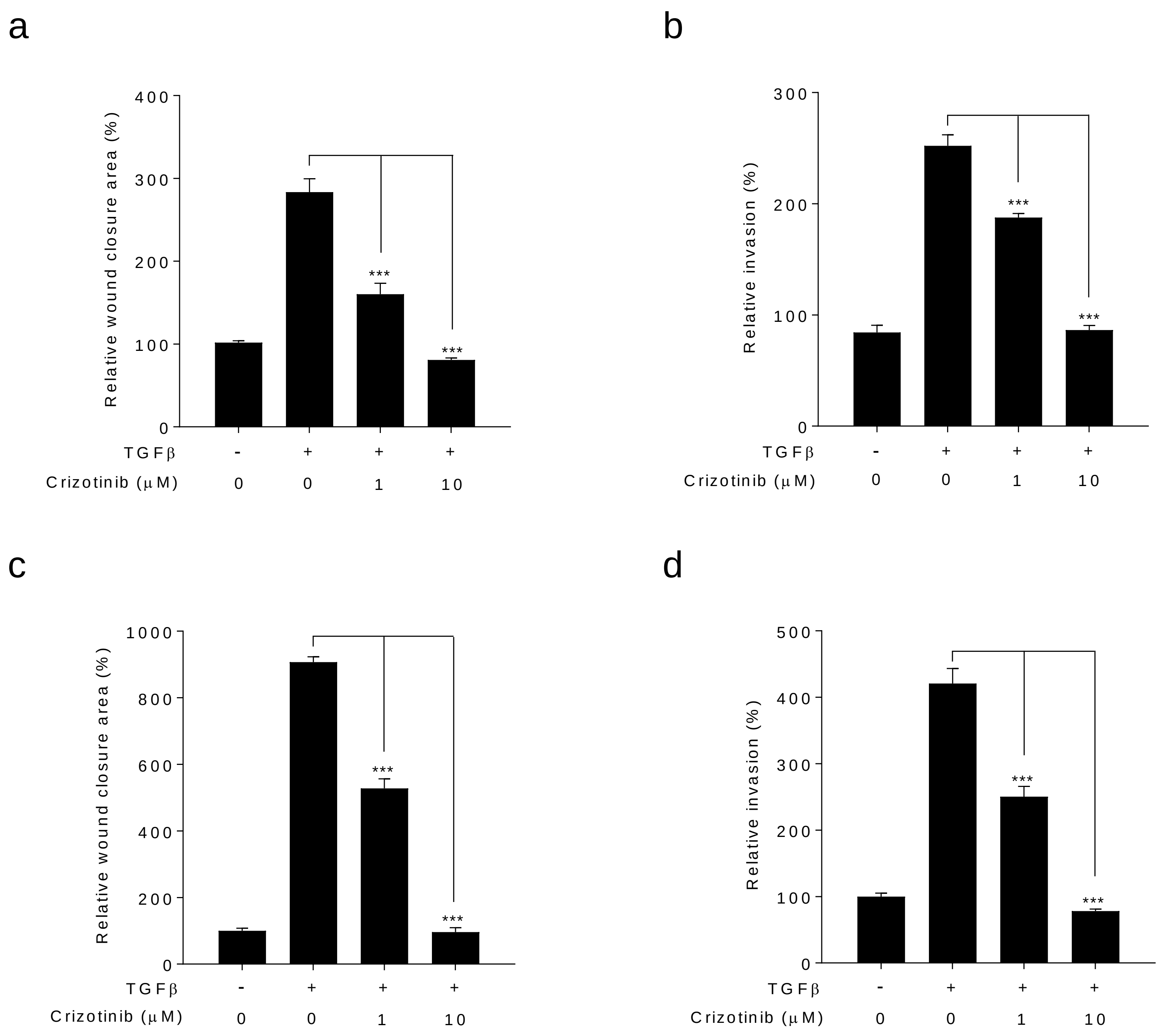

Supplementary Fig. 12

a

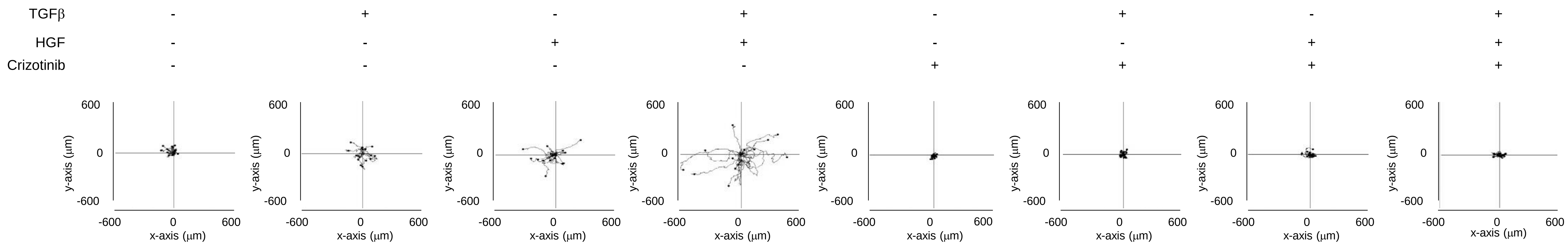

b

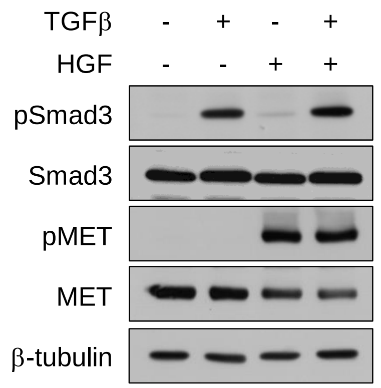

c

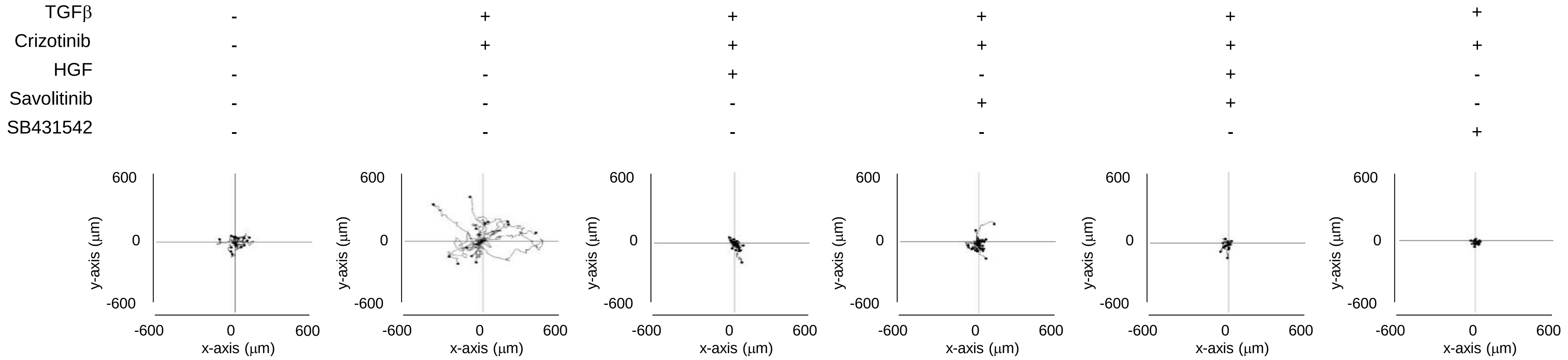

d

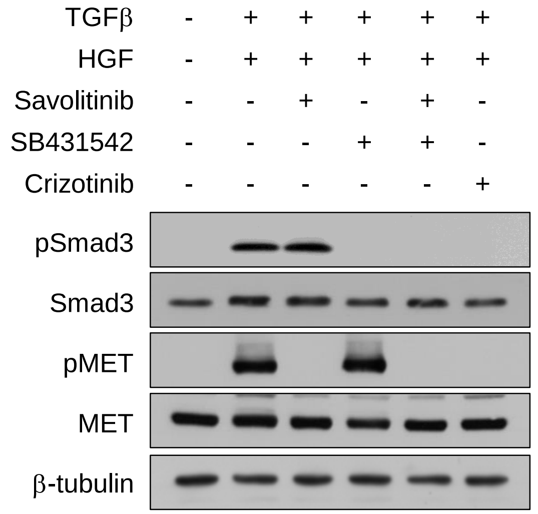

Supplementary Fig. 13

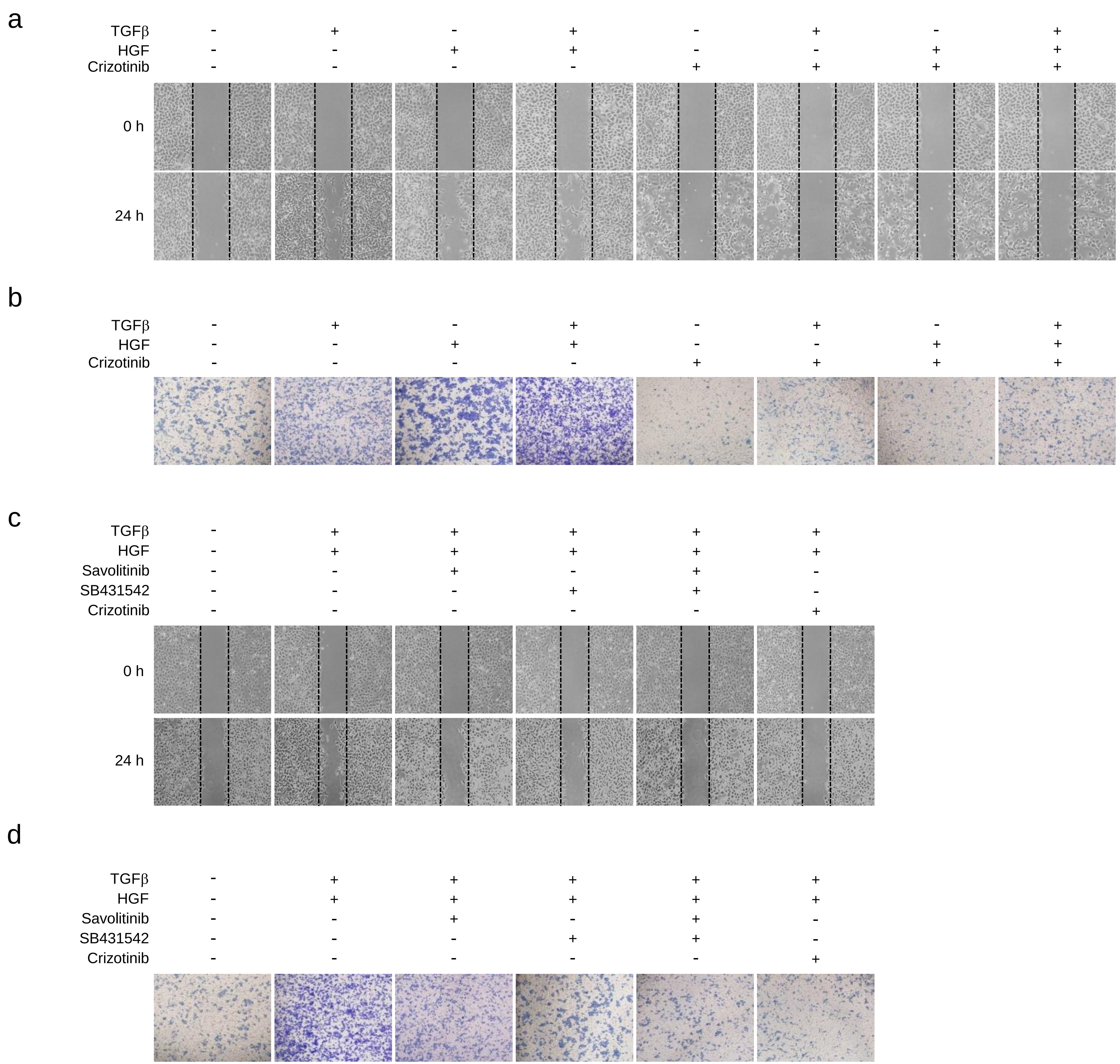

Supplementary Fig. 14

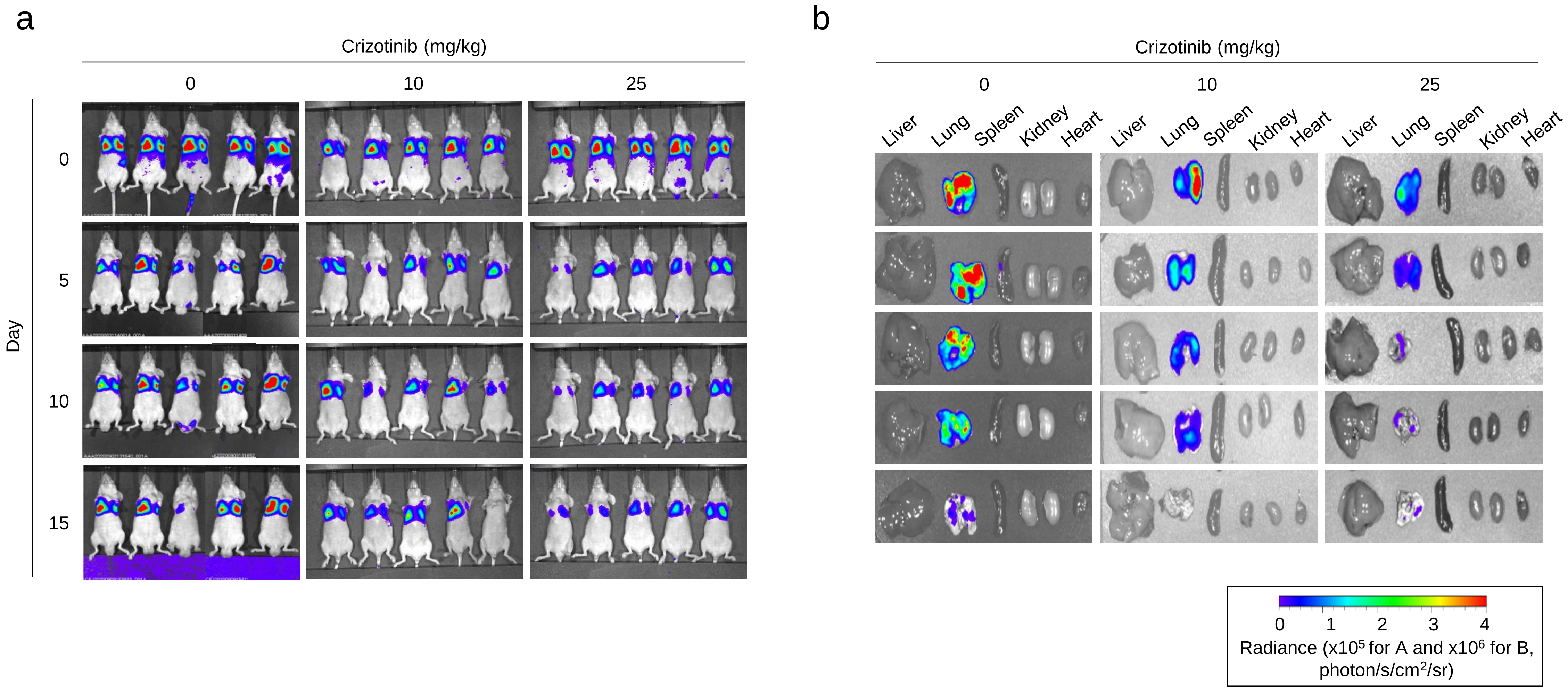

Supplementary Fig. 15

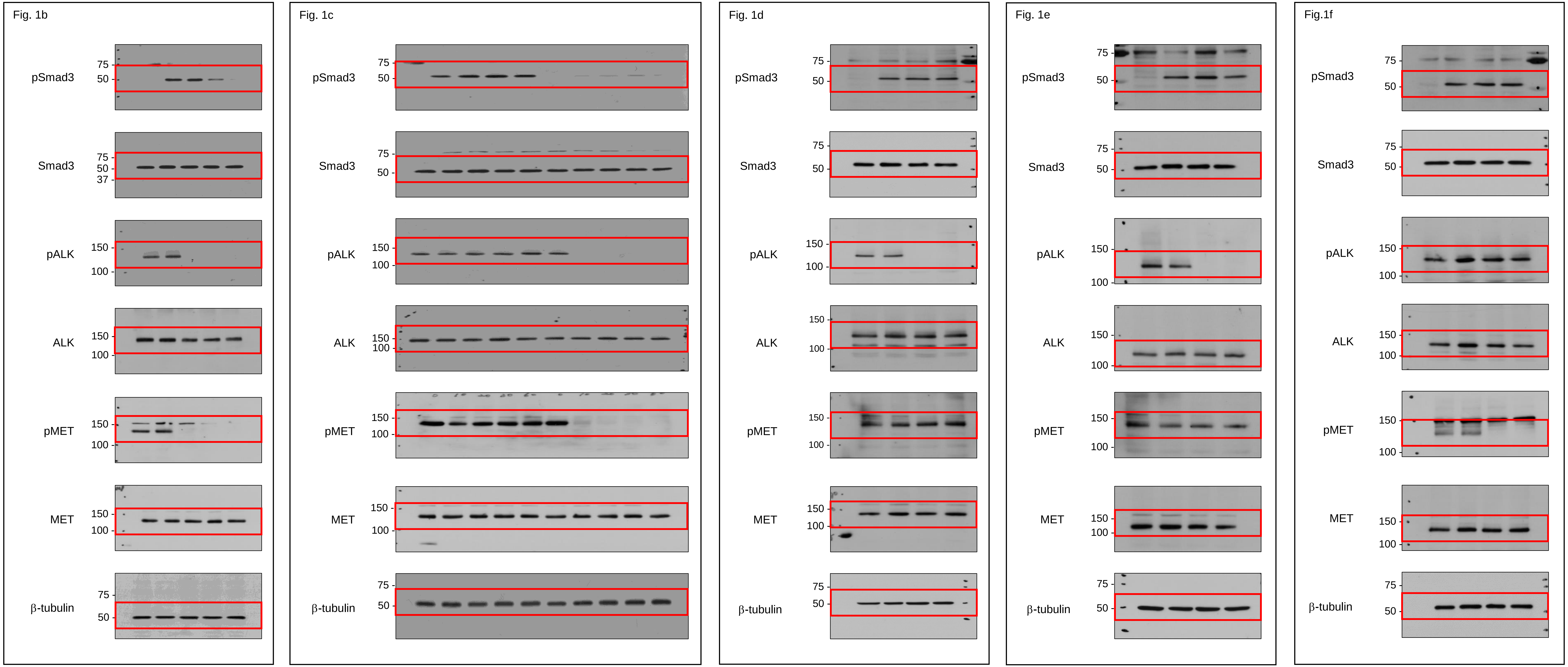

Supplementary Fig. 15

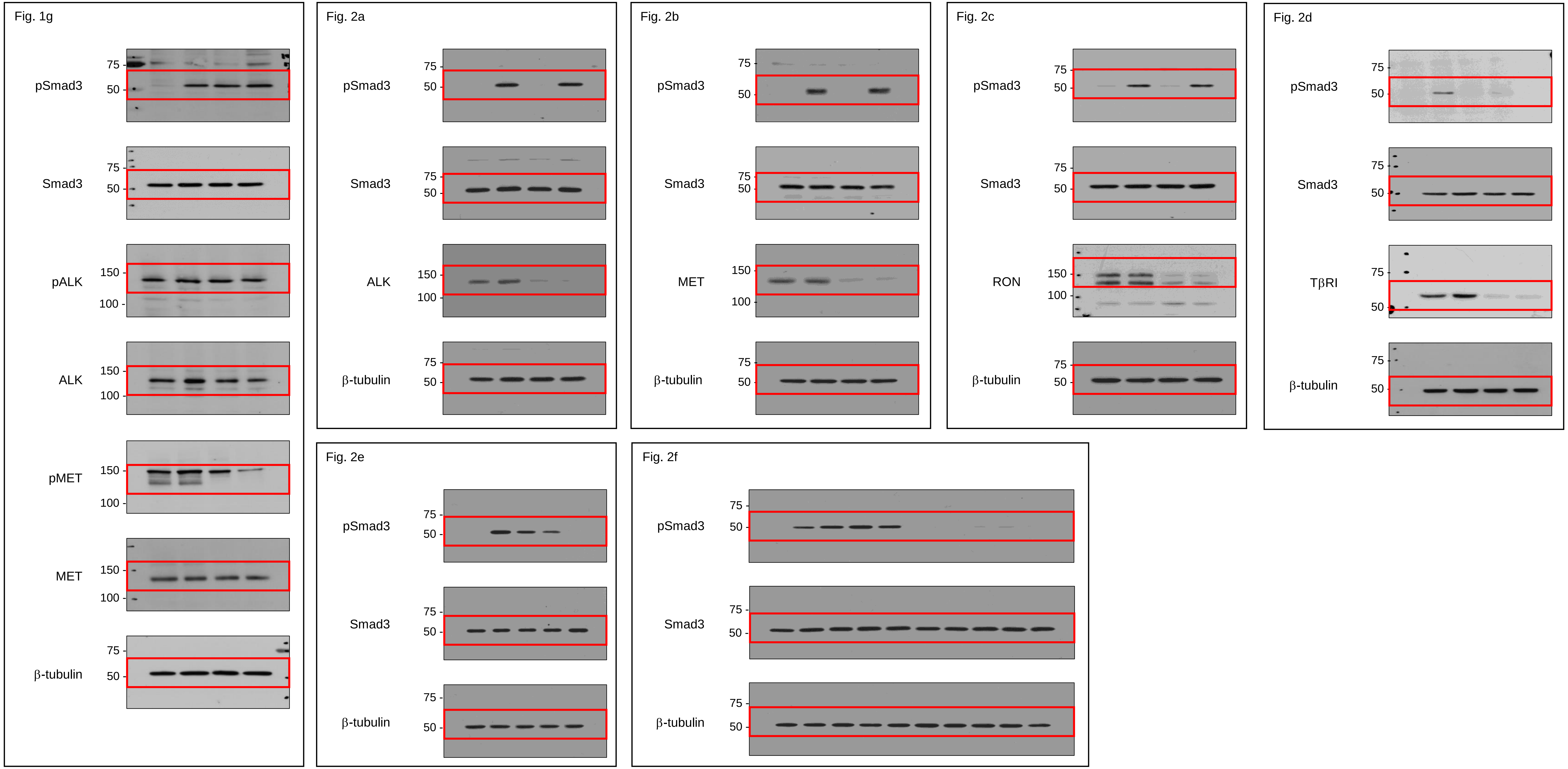

Supplementary Fig. 15

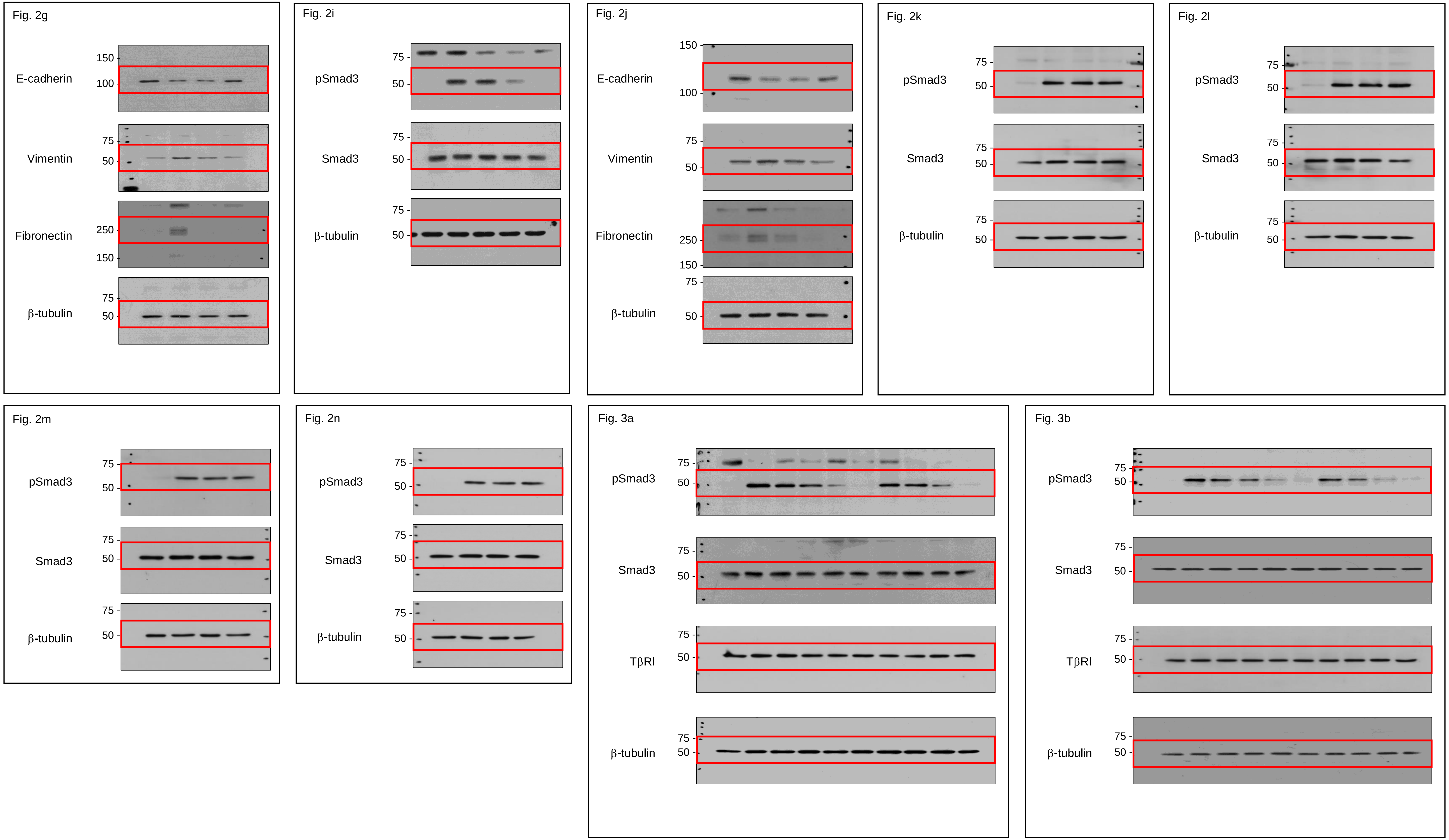

Supplementary Fig. 15

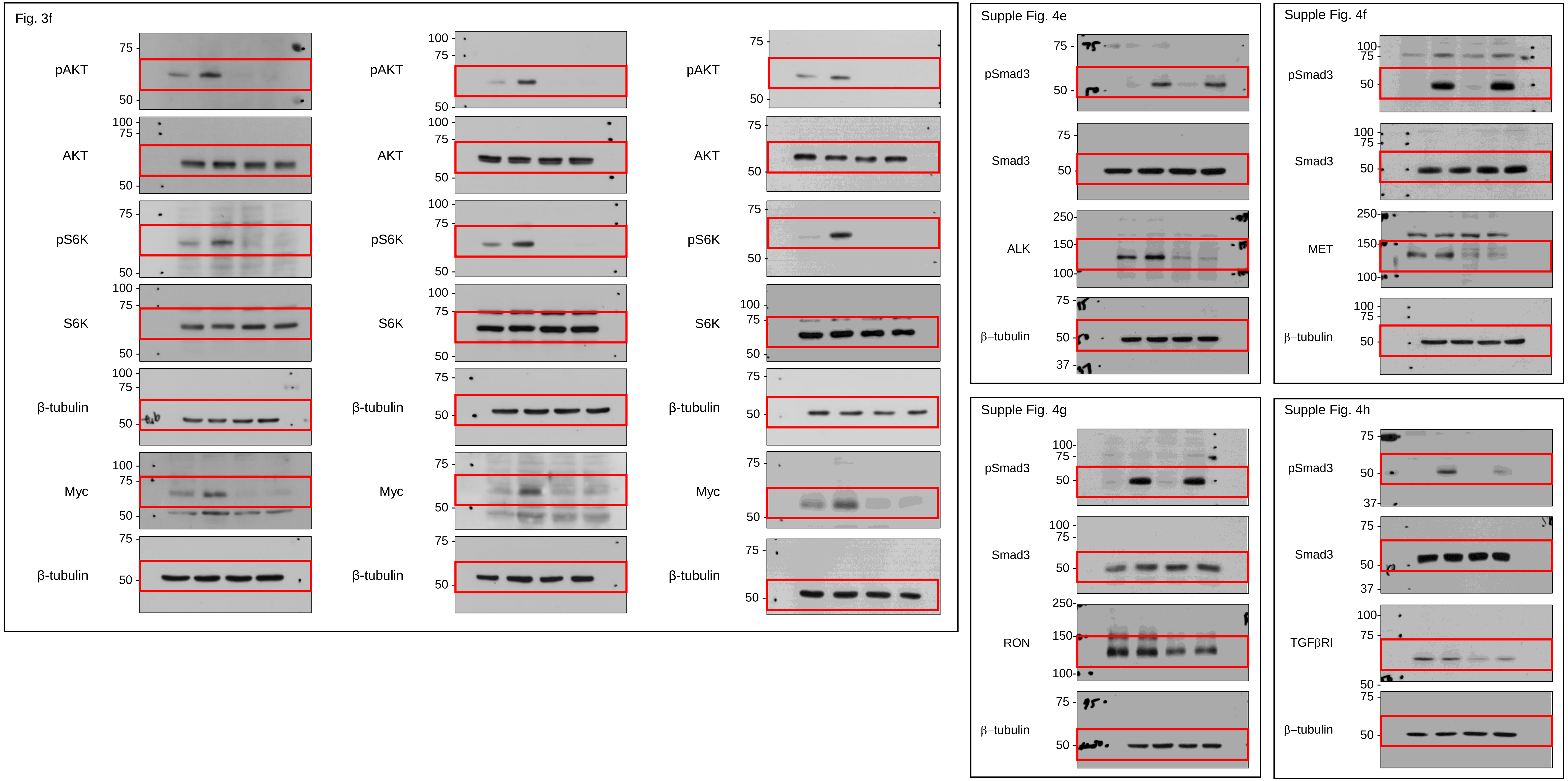

Supplementary Fig. 15

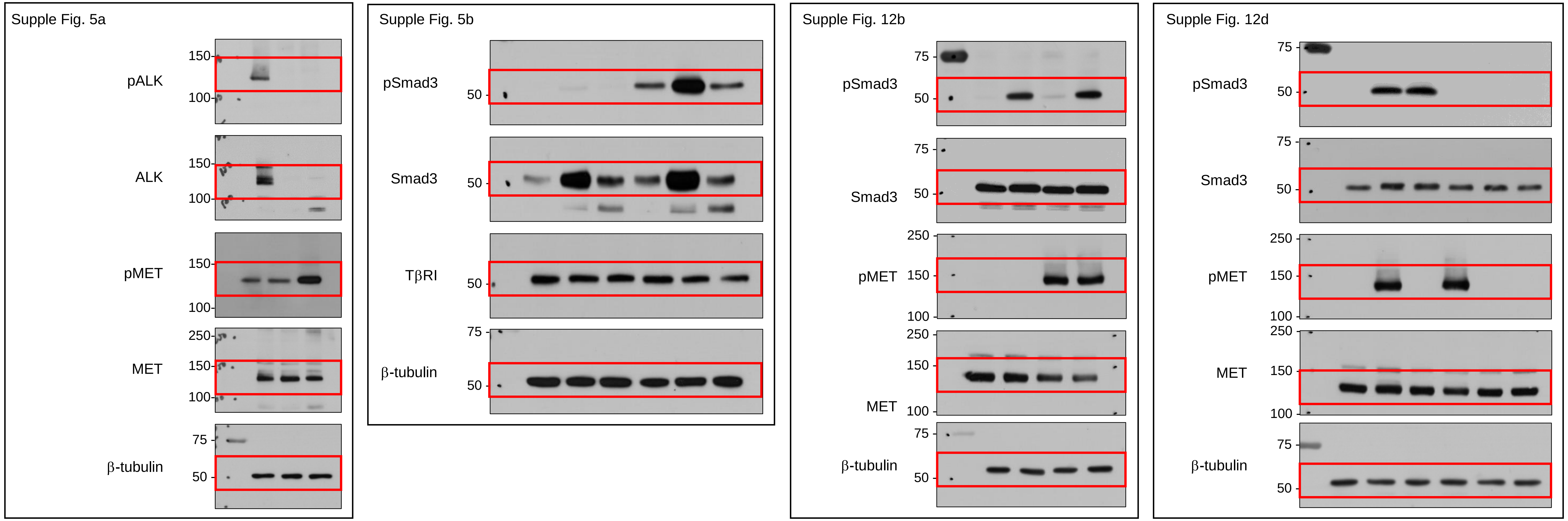

Supplementary Fig. 15

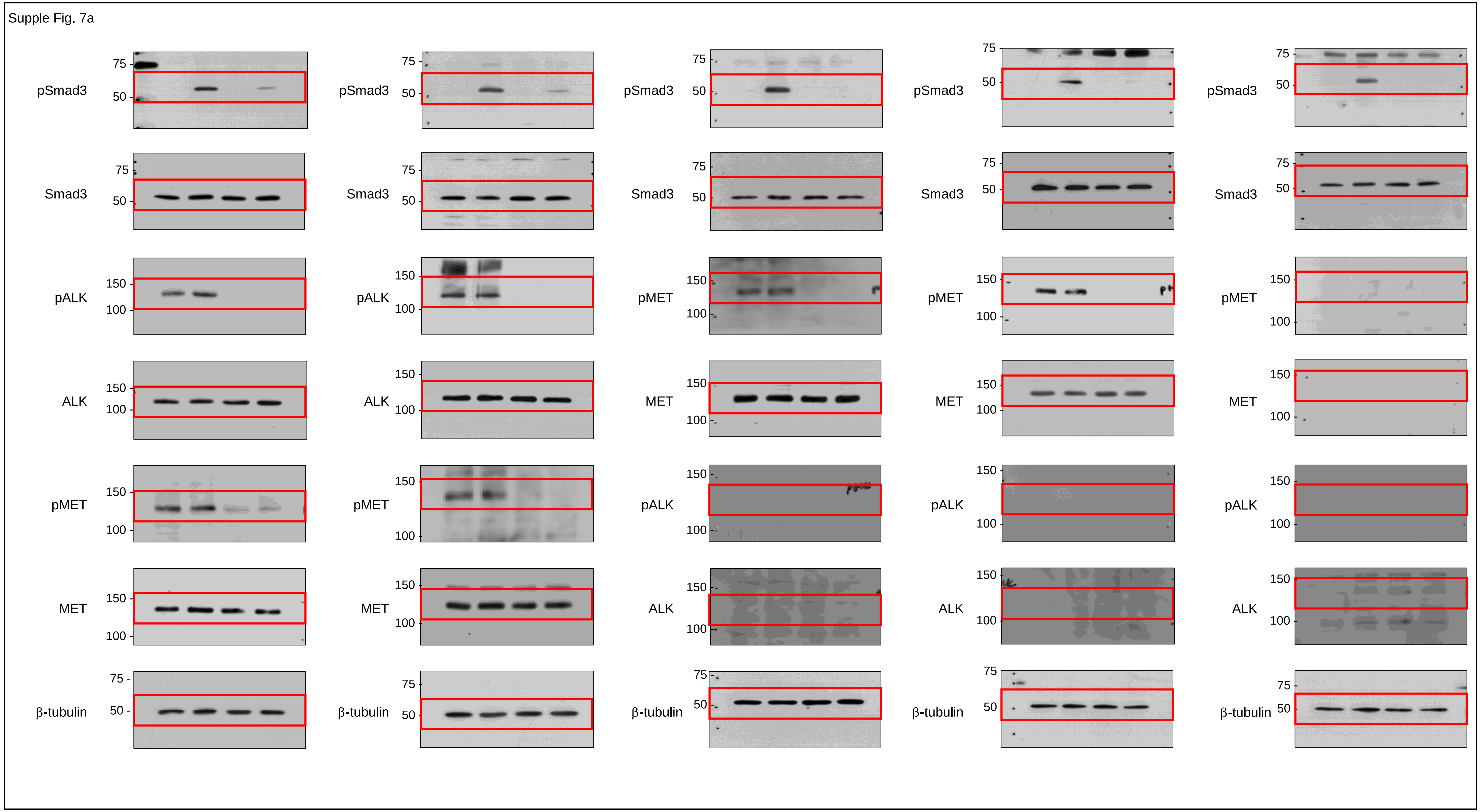

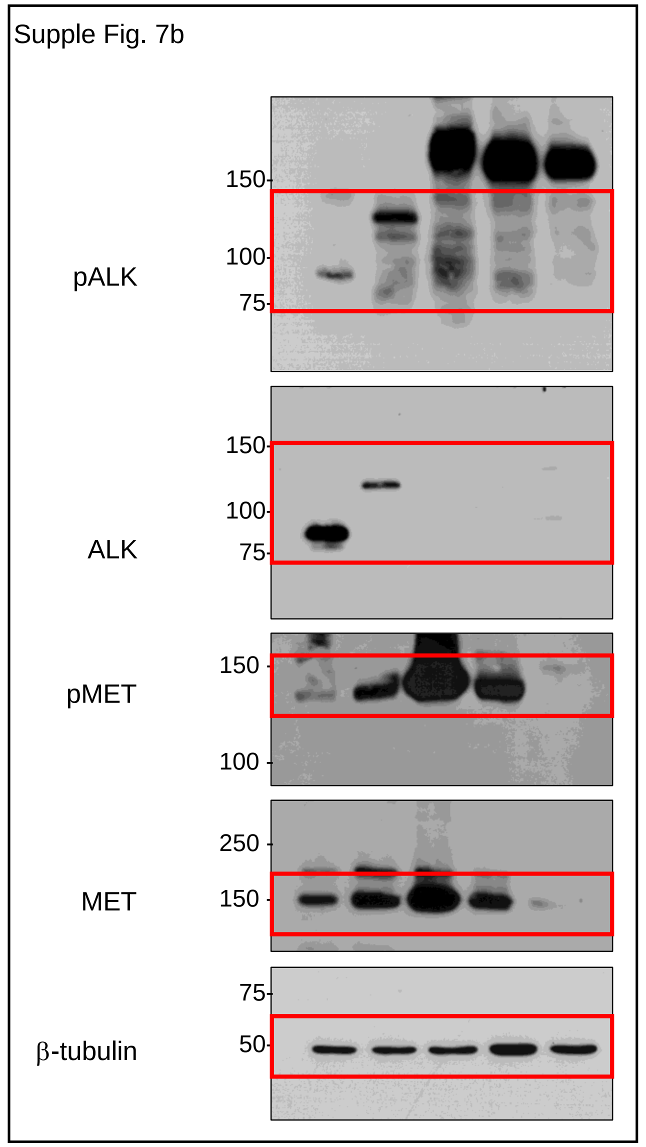

Supplement: Supplementary file 1 — Supplementary information [file 12276_2022_835_MOESM1_ESM.pdf]
